# GANs for digital art in entertainment: techniques, applications, and aesthetic evaluation

**DOI:** 10.3389/fpsyg.2026.1897981

**Published:** 2026-08-12

**Authors:** Zhang Xiaoyan

**Affiliations:** Xinzhou Normal University, Xinzhou, Shanxi, China

**Keywords:** aesthetic evaluation, AI-driven creativity, deep learning, digital art generation, generative adversarial networks

## Abstract

Artificial Intelligence has revolutionized digital entertainment, such as generating highly realistic and creative visual content, and Generative Adversarial Networks (GANs) have proven to be a pivotal technology in the synthesis and generation of images for artistic purposes. The aim of this review is to conduct a thorough and systematic study of the GAN-based digital art systems, their architectures, applications and evaluation approaches, with the objective of understanding their role in the context of contemporary creative AI research. The study uses a structured review method (PRISMA), the literature from key scientific databases, which is clearly defined and selected by inclusion and exclusion criteria that make it relevant and quality. The results demonstrate various GAN architectures such as Vanilla GAN, DCGAN, Conditional GAN, CycleGAN, StyleGAN, attention-based, and multimodal GANs applied to domains including gaming, animation, film production, virtual reality, NFT art, and music visualization. The review also highlights the important trends in the aesthetic evaluation, both objective and subjective, human-centered approaches. Overall, the study identifies some research gaps such as inconsistency of evaluation, ethical issues, computational complexity, and lack of interpretation, and suggests future trends along three main directions: hybrid generative models, real-time systems and emotionally intelligent AI for next-generation digital entertainment.

## 1 Introduction

### 1.1 Background

The digital entertainment industry has transformed dramatically with digital technologies, which offer opportunities to create rich, intelligent and immersive multimedia experiences. In the field of entertainment, AI is making its mark in several industries, including gaming, filmmaking, animation production, virtual reality, and interactive media, by optimizing creative production processes and improving the quality of entertainment products. The adoption of AI-driven systems in creative industries is becoming increasingly vital for their ability to facilitate efficient content creation, enhance creativity, and personalize user experiences. AI has advanced even more in its capabilities with the rise of computational creativity, as machines can now create art and design that can compete with that of humans.

The powerful ability of GANs to generate high-quality and realistic images through adversarial learning has established them as one of the most prominent architectures in the landscape of generative AI. Fundamentally, a GAN consists of two neural networks engaged in a minimax game: a generator that synthesizes data from latent noise, and a discriminator that learns to distinguish real samples from generated ones. This adversarial framework has driven tremendous progress in recent years, yielding impressive advances in image generation, semantic representation, and training stability. To enhance the quality of conditional image synthesis and generation diversity, Kas et al. (2024) proposed a novel method called EigenGAN, which introduces a subspace learning technique based on singular valuedecomposition. Likewise, Tian et al. (2024) presented an improved GAN approach that achieved more realistic visuals and higher image quality by incorporating optimized adversarial learning strategies.

With the advancement of computational creativity, researchers are exploring the creative potential of GANs for creative AI applications. GAN systems can learn complex distributions of artworks from a training set, and create paintings, sketches, artistic portraits, fantasy worlds, and abstract visual designs. Pal et al. (2024) investigated how training sets affect the artistic output of GAN-generated images. They showed that diversity in training sets is important to enhance the creativity and aesthetic quality of the generated images. In addition, Yildiz et al. (2024) introduced a Self-Attention GAN model combined with DenseNet architectures to improve the feature representation and create visually coherent artistic images.

Training stability and generation consistency are another significant problem in the GAN’s research. To tackle this problem, Seo et al. (2024) introduced stabilized GAN training by kernel-histogram transformation and probability mass function distance optimization that enhanced convergence performance and robustness to image generation. Moreover, He et al. (2024) proposed an introspective GAN that is capable of incremental image generation and classification by adaptive adversarial learning. The developments highlight the high potential of the GANs to create a computational creativity paradigm and catalyze AI-driven digital art production in contemporary entertainment systems.

### 1.2 Digital art in entertainment

Digital art is an important part of today’s entertainment, allowing for immersive and interactive visual experiences in various fields. For procedural environment generation, character creation, and texture design, GAN-based image synthesis is applicable in the field of gaming, and Wang et al. (2024) introduced SCGAN for distributed image generation with high efficiency for large-scale virtual worlds. GANs are used in the fields of film and animation to enhance CGI images, face synthesis and visual effects; for example, Yildiz et al. (2024) used self-attention-based GANs to boost image coherence, and Wiem and Ali (2024) proposed a new GAN called FFGAN for creating realistic frontal faces. GANs can generate realistic avatars, environments, and even virtual versions of physical assets, providing a more immersive experience for users in virtual reality and metaverse applications. Furthermore, its authenticity and stylistic flexibility make it perfect for digital art websites, like NFT or blockchain, where distinctive digital collectibles are vital. Moreover, GANs can facilitate more interactive storytelling, enabling them to produce dynamic and context-appropriate images, which can improve user engagement. Additionally, in the field of cultural heritage preservation, Yu et al. (2024) developed the ancient mural restoration model based on AGD-GAN, which showcased the application of GANs in the field of digital preservation.

### 1.3 Why GANs for art creation

GANs are very popular for digital art creation because of their capabilities of learning complex visual distributions from large-scale data sets by using adversarial training and generating realistic, aesthetically rich and semantically rich images. They can also create photorealistic portraits, artistic paintings, and abstract compositions that are visually consistent, as shown by Tian et al. (2024), who achieved enhanced GAN architectures for greater realism and fidelity, and Shang et al. (2024), who demonstrated that evolutionary multi-branch GANs can achieve high diversity and high creativity. GANs are also widely used for style transfer, transfer of artistic patterns from paintings and sketches to new images. Yildiz et al. (2024) have improved style consistency by adding a self-attention mechanism to GAN models. Also, He et al. (2024) noted that introspective GANs can further strengthen semantic consistency and incremental learning for increased visual realism, and Seo et al. (2024) demonstrated that the use of advanced normalization methods can lead to better training stability and decrease the risk of mode collapse. Moreover, Pal et al. (2024) highlighted that diversity in datasets and adversarial learning are crucial in boosting creativity and artistic output, making GANs an effective and valuable tool for creative and contemporary digital art creation by artificial intelligence.

### 1.4 Motivation for systematic review

Although there have been several extensive surveys on GANs and AI-generated content, there is a lack of literature that specifically examines the technical architecture, aesthetic assessment, and media psychology of GANs and AI-generated content. Habib et al. (2024) conducted a comprehensive review of the advancements in GANs for text-to-image generation, with a particular focus on architectural developments and benchmark comparisons. In a similar vein, Cao et al. (2023) and Liu et al. (2023) studied AI-generated content (AIGC) and computational creativity mainly from a computer science perspective, emphasizing model scalability and fidelity of generation. Epstein et al. (2023) discussed the philosophical and cognitive aspects of machine creativity, but provided little discussion of the current GAN architectures and their use in entertainment ecosystems.

They are useful but often aesthetic assessment is a secondary issue, and the findings of these surveys do not often incorporate the findings of media psychology or audience-centred perception frameworks. The present review has four major contributions. First, it presents a multi-layered taxonomy that both categorizes GAN systems by architectural design, generation techniques, application areas (gaming, animation, film, VR, NFTs), and evaluation approaches, thus offering a more comprehensive perspective than previous single-dimensional surveys. Second, it emphasizes the aesthetic evaluation, systematically comparing objective metrics (FID, IS, PSNR, SSIM) and subjective, human-centered evaluations, thus closing the technical and perceptual quality gap. Third, it explicitly relates art production by GAN to media psychological paradigms such as cognitive load, processing fluency and emotional valence tracking, providing a psychological perspective that has not been well addressed in previous literature. Fourth, it is strictly themed and focused on digital entertainment and creative uses of AI, with a conscious effort to avoid topics from industrial, medical, and purely technical areas, in order to maintain conceptual coherence and relevance for the audience of the journal. This review combines these viewpoints to create a comprehensive framework that not only documents technological advancement but also sheds light on the role of GANs in today’s digital culture.

The objective of this Systematic Review is to provide a comprehensive review of the existing literature on digital art with the GAN approaches and to consolidate all the findings from the different studies and create a unified framework. It also aims to enable a comparison of different GAN architectures, applications, and testing methods to gain insight into their pros and cons. Moreover, the drawbacks of using GANs for the generation of art are addressed in the review, including training instabilities, inaccurate evaluations, and ethical concerns. Finally, it seeks to highlight key research gaps to inspire future research that would help create more powerful and interpretable generative models for digital art and creative-AI.

### 1.5 Objectives

- To critically review and combine previous studies of GAN-based digital artwork and creative AI systems.
- To classify and review the important GAN architectures for image and art generation.
- To analyze how GANs can be used in various fields like gaming, animation, film, VR, and digital art.
- To assess and compare objective and subjective techniques for art assessment of GAN-generated art.
- To understand the key issues and limitations of current creative systems based on GANs.
- To highlight future research areas of GANs and their applications in the ethical context, interpretability and performance.

### 1.6 Contributions of the review

This review makes its contribution by providing an in-depth taxonomy of the GAN architectures used to create digital art, a structured comparison of the models used, applications and evaluation methods. It also provides an overview on objective and subjective aesthetic evaluation methods that may help to understand the notion of artistic quality. Furthermore, the study identifies key open challenges and research areas for creative AI systems leveraging GANs, including technical challenges, evaluation difficulties, and ethical considerations, offering insights into potential future research avenues.

## 2 Background and fundamental concepts

### 2.1 Artificial intelligence in creative systems

Creative systems using AI cover the use of AI to create digital content that is interactive and artistic, using computational creativity and generative models. According to Xiang (2023) the GAN-based models can learn artistic styles and create aesthetically pleasing images, and Zhang H. et al. (2023) reported that the disentangled generative adversarial networks can improve the aesthetics of images. Furthermore, Ma et al. (2025) proposed the interactive art production system Time Media, focusing on the interaction between humans and AI. In conclusion, significant progress in GANs, transformers, and diffusion models has revolutionized AI-based content generation in the realm of digital art and entertainment.

### 2.2 Deep learning for image synthesis

Deep learning is a key technology in image synthesis that automatically learns hierarchical visual features for generating realistic images. Yildiz et al. (2024) used CNNs with self-attention mechanisms in DenseNet to enhance the synthesis quality, and autoencoders enable the reconstruction and manipulation of images through learning latent representations. In addition, diffusion-based models have continued to make significant contributions, such as SwinV2-ImageN (Li et al., 2024) and DiffiT (Hatamizadeh et al., 2024) for achieving more realistic and semantically consistent images, and scalable high-resolution diffusion transformer models developed by Crowson et al. (2024). In general, CNNs, autoencoders, diffusion models and transformers improve the performance of contemporary deep learning–based image synthesis systems.

### 2.3 Fundamentals of GANs

GANs are a type of deep learning model that works on the principle of adversarial training and comprise two parts: a generator that generates synthetic images from noise, and a discriminator that classifies whether an image is real or fake. It works by iterative adversarial training, which essentially involves the two networks being trained in turn until they get better and better, leading to the creation of extremely realistic images. The model is trained on a minimax optimization strategy, where the generator learns to fool the discriminator, while the discriminator improves its classification accuracy, resulting in good learning of complex data distributions and high-quality image synthesis. The mathematical objective function of GANs is given by:

$$\begin{array}{l} \min_{G}\max_{D}V(D,G)=E_{x∼p_{\text{data}}(x)}[\log D(x)]\\ +E_{z∼p_{z}(z)}[\log(1-D(G(z)))] \end{array}$$ (1)

where: G represents the generator network, D represents the discriminator network, $x∼p_{\text{data}}(x)$ denotes real data samples, $z∼p_{z}(z)$ represents random noise vectors, G(z) indicates generated synthetic images and D(x) represents the probability that image x is real.

The first term maximizes the discriminator’s accuracy at correctly classifying real images, and the second term minimizes the discriminator’s ability to detect the generated images. This optimization process is continued until the generator is able to generate images that are sufficiently realistic for the discriminator so that it can no longer reliably differentiate between them and real images.

The quality of generation, semantic representation, and training stability of recent GAN advancements has greatly improved. Sauer et al. (2023) proposed StyleGAN-T for rapid text-to-image generation at high resolution, showing the potential of GANs for high-resolution artistic image generation. Additionally, Kang et al. (2023) improved GAN architectures in text-to-image synthesis by enhancing semantic conditioning and large-scale adversarial learning. Wang et al. (2023) designed a MemoryGAN framework based on GAN generators as heterogeneous memory modules for compositional image synthesis. The progress made in this field underscores the promise and growing relevance of GANs in today’s digital art-making and computational creativity.

### 2.4 Evolution of GAN architectures

The development of GAN architectures has greatly improved the image synthesis capabilities, yielding high-quality, stable, and controllable output images. Early foundational models such as the DCGAN further improved the learning of spatial features, while cGAN (Rosenberg et al., 2015) added conditional and controllable generation. For better compositional synthesis, Wang et al. (2023) proposed MemoryGAN, and Sauer et al. (2023) presented StyleGAN-T, a model for text-to-image generation. In total, these developments with CycleGAN, the variants of StyleGAN, BigGAN, and SAGAN have increasingly enabled more realistic image generation, semantic consistency, and artistic skills to GAN systems.

### 2.5 Digital art generation workflow

The digital art generation workflow for creating realistic and creative digital artworks involves several steps, such as dataset collection, preprocessing, training, image generation, post-processing, and evaluation. Pal et al. (2024) emphasized the significance of the diversity of datasets for enhancing artistic quality. Seo et al. (2024) proposed stabilized GAN training for achieving better convergence, and Sauer et al. (2023) developed improved text-to-image synthesis using StyleGAN-T. These stages are important in the overall process of high-quality generation of digital artworks in terms of FID and IS.

## 3 Systematic review methodology

The method used in this study is systematic literature review that can be used to conduct a systematic study in the fields of digital art generation and creative applications using GANs. The methodology used is aimed at transparency, repeatability and rigor in selecting and evaluating pertinent studies. The study takes the reader through the carefully selected review process, which gathers the findings of 65 high-quality scientific papers concerning the GAN architectures, image synthesis techniques and applications, such as games, film, virtual reality, and digital art. Studies exclusively focused on non-art domains (medical imaging, industrial anomaly detection, sonar engineering) were excluded to maintain thematic consistency with the journal’s focus on media psychology and digital aesthetics. Studies are identified, screened, assessed for eligibility and included in the review systematically using the PRISMA framework to ensure consistency of the methodology followed across the review process.

### 3.1 Review protocol

The review protocol follows guidelines set in PRISMA 2020, a standard protocol for systematic literature reviews. This protocol outlines the research aim, eligibility criteria, search strategy and analysis techniques of the study. The main focus is to explore GAN-based architectures and their development in digital art generation, both technically and application-oriented. The protocol outlines each step of the review process systematically, minimizing the chances of bias and increasing the chances of the study being reproduced. A systematic process is employed, such as literature identification, screening, eligibility evaluation and final selection of studies for analysis.

#### 3.1.1 Research question and PICOS framework

To follow the PRISMA 2020 guidelines and ensure methodological rigor, transparency, and alignment with the framework, the primary research question of this systematic review was formulated using PICOS (Population, Intervention, Comparator, Outcomes, and Study Design) framework. This systematic process clearly establishes the scope, boundaries and evaluative focus for the review, which helps to reduce selection bias and thematic consistency. The main research question for this study is: What are the impacts of different GAN architectures on digital art creation, usage, and visual perception in entertainment and creative AI sectors?

The specific PICOS criteria applied for study selection and analysis are defined as follows:

- Population (P): Studies on digital art generation, computational creativity and media entertainment applications (gaming, animation, film production, virtual reality, and NFTs) with empirical studies and computational models. Studies that were entirely outside the focus of media psychology and digital aesthetics, such as work in non-art fields like industrial anomaly detection, medical imaging, or just structural engineering, were not included, as they did not fit the thematic focus.
- Intervention (I): Application, modification and development of Generative Adversarial Network (GAN) architectures. This involves basic models (Vanilla GAN, DCGAN) and advanced models (Conditional GAN, CycleGAN, StyleGAN, attention-based, and multi-modal GANs) used for image synthesis and artistic generation.
- Comparator (C): Comparative evaluation of different GAN architectures, baseline generative models and, if applicable, emerging generative paradigms (Diffusion models, Transformer-based architectures) to situate GANs in the context of generative AI. The main outcomes are related to the aesthetic and perceptual evaluation of the generated art, including objective metrics (Fréchet Inception Distance [FID], Inception Score [IS], Peak Signal-to-Noise Ratio [PSNR] and Structural Similarity Index Measure [SSIM]) and subjective, human-centred ones (user preference, expert artistic evaluation, cognitive load and creativity perception). Secondary outcomes are technical parameters like training stability, resolution of the images and computational efficiency.
- Study Design (S): Empirical studies, experimental evaluation, and systematic surveys published in high impact journals and conferences from 2015–2026 that have been peer-reviewed. Included studies should be clearly methodologically structured, make use of clearly formulated data sets and present clear evaluation parameters.

### 3.2 Research databases

Relevant literature was gathered from various recognized academic databases in order to achieve a wide coverage of the literature on the topic of GAN. The databases selected are IEEE Xplore, ScienceDirect, SpringerLink, ACM Digital Library, Scopus, Web of Science and Google Scholar. These databases have been selected because they provide good coverage of peer-reviewed journals and high-impact conference proceedings for artificial intelligence, machine learning and computer vision. The use of multiple databases provides diversity of research perspectives and decreases publication bias. The interdisciplinary, engineering-based sources can provide a broad view on the GAN application for digital art and creative industries.

### 3.3 Search strategy

A structured search strategy was employed to conduct search using keywords and Boolean operators, to identify relevant studies. The search phrases used encompassed ‘GAN’ OR ‘Generative Adversarial Network,’ and ‘Digital Art,’ ‘AI Art,’ ‘Image Synthesis,’ and specific application terms like ‘Gaming,’ ‘Film,’ ‘Metaverse. In architectures such as StyleGAN, CycleGAN and Conditional GAN, additional fine-grained queries were used. The time period was limited to papers published from 2015 to 2026, with more weight given to the more recent studies (2023–2026) because of the important developments in generative modelling over this period. This approach provided both breadth and relevance to the literature selected.

### 3.4 Inclusion criteria

For the purpose of selecting high-quality and relevant studies, the inclusion criteria were determined. We considered only peer-reviewed journal articles and high-quality preprints in English. Studies were selected if they involved image generation using GAN, artistic synthesis or creative use in the entertainment, games, virtual reality, and digital art sectors. Also, research published from 2015 to 2026 was considered to reflect both landmarks and recent advances in the field of GAN. Only empirical studies, with clear methodology, clear use of the data and evaluation metrics, were used in order to ensure reliability in the analysis.

### 3.5 Exclusion criteria

The exclusion criteria were used to exclude irrelevant, low-quality or non-contributing studies from the review process. Studies which used methods other than the GAN approach and those that were not directly related to creative and visual applications were excluded. Articles that are not peer-reviewed, opinion pieces or studies without method or experimental evaluation were also excluded. The data from the multiple databases were eliminated to prevent duplication. In addition, only studies that focused only on non-art domains like purely industrial anomaly detection or non-related biomedical applications were not taken into account in order to maintain the thematic consistency of this review.

### 3.6 PRISMA flow diagram

The process of study selection was performed using the PRISMA flow diagram, which comprised four phases: identification, screening, eligibility, and inclusion. About 210–250 records were taken from various databases in the identification phase. About 170 studies were left after duplicate data were removed for initial screening. The titles and abstracts were screened and studies that were not relevant were excluded. The full text articles were then analysed for methodology and the relevance to the selected topic was determined to reduce the data set at the eligibility phase. Finally, 65 studies were selected, following the PICOS framework and exclusion criteria that excluded studies from other fields of non-art, like medical imaging, industrial anomaly detection, and sonar engineering. The taxonomy, classification and application analysis presented in this review is taken into account based on these studies. To make sure the review was both theoretically and empirically comprehensive, 65 studies were included in the final review, but Table 1 breaks down the 61 core empirical studies that explicitly provided data on the datasets, applications, and metrics. The other 4 studies are general survey papers and theoretical papers (Cao et al., 2023; Epstein et al., 2023; Hu et al., 2026; Zhang et al., 2025) that helped develop the conceptual taxonomy but did not provide any stand-alone empirical metrics.

**Table 1:** Comparative analysis of GANs, diffusion models, and flow matching for digital art generation.

Criterion	GANs	Diffusion models	Flow matching
Training stability	Low (Prone to mode collapse; requires delicate balancing of generator/discriminator)	High (Optimizes a single, well-defined noise-prediction objective)	High (Learns a continuous transport map with stable convergence)
Inference speed	Very Fast (Single forward pass; ideal for real-time rendering)	Slow (Requires 20–100 iterative denoising steps)	Moderate to Fast (Achieves high quality in significantly fewer steps than diffusion)
Image quality and detail	High (Excellent realism, but may exhibit minor artifacts or blurring)	Very High (Superior fine-grained detail, coherence, and photorealism)	High (Comparable to diffusion models, with improved sharpness in fewer steps)
Semantic alignment	Moderate (Struggles with complex, multi-concept textual prompts)	Very High (Exceptional prompt adherence, especially when CLIP-guided)	High (Rapidly improving with advanced conditioning mechanisms)
Output diversity	Moderate (High risk of mode collapse limiting variety)	Very High (Generates highly diverse and novel compositions)	High (Encourages diverse sampling paths)
Controllability	Very High (Precise, fine-grained manipulation of latent space attributes)	Moderate (Improving rapidly via tools like ControlNet, but less intuitive than GAN latent spaces)	Moderate (Emerging techniques for conditional generation)
Computational cost	Moderate (Training can be expensive, but inference is highly efficient)	Very High (Both training and multi-step inference are extremely resource-intensive)	High (Training is expensive, but inference is more efficient than diffusion)
Real-time suitability	Excellent (Widely used in gaming, interactive media, and live installations)	Poor (Too slow for real-time without aggressive distillation or consistency models)	Good (Strong potential for real-time interactive systems with further optimization)
Video/temporal generation	Good (Established architectures for frame interpolation and temporal consistency)	Challenging (Maintaining temporal coherence across frames remains a significant hurdle)	Emerging (Limited empirical validation in large-scale video tasks)
Community ecosystem	Mature but declining in mainstream text-to-image focus	Very Active (Dominates current research; e.g., Stable Diffusion, DALL-E, Midjourney)	Early stage (Rapidly growing academic and industrial interest)
Primary art use cases	Real-time generation, character design, interactive art, video synthesis, style transfer	High-quality text-to-image, complex compositional art, commercial digital art platforms	Fast high-quality generation, interactive creative systems (future potential)
Key limitations	Mode collapse, training instability, limited open-domain semantic control	Slow inference speed, massive computational overhead, less direct latent controllability	Early developmental stage, limited large-scale empirical validation in art domains

### 3.7 Quality assessment

All of the studies selected was assessed for methodological rigour using a structured quality assessment framework. The assessment was done on the following criteria: methodological clarity, experimental verification, repeatability and transparency of the data set. Each study received a ranking from 0 to 2 for each criterion, with a total of 8 possible points. Studies with well-designed experiments, well-defined GAN architectures, and well-defined datasets were awarded more points. This process allowed for the inclusion of only high-quality and reliable studies, enhancing the credibility and robustness of the findings of the review.

### 3.8 Data extraction strategy

Systematically, relevant data from selected studies were extracted following a standardized data collection strategy. The attributes that were extracted were the type of GAN, the type of dataset, the application domain, the evaluation metrics, the key results, and the reported limitations. This systematic procedure helped to ensure the uniformity of comparing studies and helped to develop taxonomy and classification schemes. It also assisted in the discovery of patterns in GAN architectures, enhancements, and adaptations for specific applications. Data collected were used to analyze the evolution of GANs in digital art generation and to map technique-dataset-application domain relationships.

## 4 Taxonomy of GAN-based digital art generation

GANs taxonomy provides a structured overview of the various GAN models and their progress from the initial generation of GANs to the more sophisticated models. It classifies the GANs according to the architecture (Vanilla GAN, DCGAN, cGAN, CycleGAN, StyleGAN, and attention-based GANs), generation techniques, style transfer techniques and applications (text-to-image synthesis, high-resolution image generation, 3D generation, and interactive/emotion-aware generation). This classification also reflects their usage in the datasets and evaluation methods, giving a clear understanding of the organization, development and application of GAN models in various artificial intelligence (AI) and digital art generation tasks.

### 4.1 Architecture-based classification

The architecture-based classification of GANs divides them into basic GANs, such as Vanilla GAN and DCGAN, advanced GANs, such as cGAN, CycleGAN, StyleGAN, attention-based, and multi-modal GANs. This classification emphasizes the role that architectural improvements play in boosting the quality of the generated images, the stability of the training process, and the ease of controlling the GAN, thereby significantly enhancing its ability to generate images and transform them into new forms.

#### 4.1.1 Vanilla GAN

The Vanilla GANs consist of a generator and a discriminator that are trained to generate realistic synthetic images by playing an adversarial game against one another. The generator generates images from random latent vectors, and the discriminator tries to classify the generated images as real or fake. This simple model laid the foundation for later GAN models. To enhance the training of GANs and the quality of generated images. Wang et al. (2024), in turn, proposed a semi-centralized GAN (SCGAN) framework for collaborative distributed image generation systems, which extends the classical GAN to a collaborative distributed image generation system. In artistic image generation, Vela et al. (2023) have improved the quality of image synthesis by employing cyclic generative strategies and top-k training methods, which not only yield an improved image quality but also maintain the essential adversarial mechanism of Vanilla GANs.

#### 4.1.2 DCGAN

DCGANs incorporate convolutional neural networks into GAN to enhance feature extraction and spatial representation learning. Instead of fully connected layers, DCGAN uses convolutional and transposed convolutional layers, which leads to more stable training and higher quality of image generation. Xin (2023) proposed an improved DCGAN architecture for fast and high-resolution image generation by optimizing convolutional feature learning and generator efficiency. To enhance low-light images. Shatri et al. (2024) have tested DCGAN models for handwritten music notation synthesis and shown the advantage of convolution-based GAN architectures in structured image generation.

#### 4.1.3 Conditional GAN

The standard GAN model is extended to Conditional Generative Adversarial Networks (cGANs), where auxiliary information like labels, semantic attributes or text descriptions is added to both the generator and discriminator. This conditioning mechanism allows for controlled and targeted generation of images. To enhance semantic consistency and controllability in conditional text-to-image GANs, Zhang and Schomaker (2024) explored the issues of latent space optimization and interpretability. Hu et al. (2019) suggested a variational conditional GAN model for latent variable modelling of controllable fine-grained image generation. Recently, Zhang and Schomaker (2024) studied latent space optimization methods for conditional text-to-image GANs to improve the semantic controllability and interpretability of multimodal artistic image generation systems.

#### 4.1.4 CycleGAN

CycleGAN architectures have been broadly applied to unpaired image-to-image translation tasks, when there is no explicit correspondence between source and target images. The architectures are based on cycle consistency loss, such that the consistency of a structure is maintained in the transformation of style. Zhao (2025) applied CycleGAN-based style transfer techniques in visual communication design, demonstrating their effectiveness in artistic and creative image transformation applications. Furthermore, Nigam et al. (2025) introduced Frequency Distributed CycleGAN (FDCGAN), a framework that leverages frequency-aware mechanisms for learning latent representations in image translation. In addition, Shatri et al. (2024) compared CycleWGAN architectures for synthesizing handwritten music and emphasized that cyclic consistency is crucial for maintaining structural image characteristics.

#### 4.1.5 StyleGAN family

The StyleGAN family presents style-based generators that can generate high-resolution and very realistic images via adaptive normalization and latent style manipulation. These architectures can greatly enhance the photorealism, controllability, and diversity of image generation tasks. Sauer et al. (2023) presented StyleGAN-T that integrates transformer-based text understanding and StyleGAN architectures to enable rapid and scalable text-to-image generation. Sauer et al. (2022). To achieve this, (2022) introduced StyleGAN-XL to apply to large and diverse datasets to enhance synthesis stability and visual fidelity.

#### 4.1.6 Attention-based GANs

Attention-based GANs involve self-attention, cross-attention, and channel attention mechanisms, which help to model long-range dependencies and contextual relationships within images. These attention mechanisms help to capture semantic consistency, preserve details of objects, and capture context learning. Xu et al. (2018) introduced AttnGAN, which is based on attentional mechanisms, the idea is to focus on the important textual features when synthesizing images. To enhance anime image generation, Lu (2024) introduced the hybrid self-attention technique (USE-CMHSA-GAN). Likewise, Tang et al. (2025) presented enhanced multi-scale cross-attention models for person image generation in order to facilitate the correspondence of image features between different regions. In the field of few-shot plant disease image generation, Wang et al. (2026) utilized the CAFE-GAN, combining CLIP-projected representations with attention-aware generation and multi-scale discrimination for semantically coherent image synthesis.

#### 4.1.7 Multi-modal GANs

Multi-modal GANs generate contextually relevant images using multiple modalities like text, semantic embedding and image. Such architectures are especially crucial for tasks like generating digital art and text-to-image synthesis. Kang et al. (2023) suggested large-scale GAN architectures for text-to-image synthesis, greatly enhancing semantic alignment and enriching visual diversity in created artistic content. To enable scalable image synthesis with multimodal inputs, Sauer et al. (2023) added text-based encoding into StyleGAN-T using a transformer. Moreover, Xu et al. (2018) designed AttnGAN, a fine-grained text-conditioned image generation system on the basis of word-level attention mechanisms. Furthermore, Zhang and Schomaker (2024) investigated latent space optimization techniques for conditional text-to-image GANs, enhancing semantic controllability and interpretability in multimodal artistic image generation systems.

### 4.2 Image synthesis techniques

Image synthesis techniques in GANs refer to the procedure for creating images from input representations such as random noise or semantic information. These methods involve generating images from noise, such as converting a latent vector to a realistic image, or semantic synthesis, which guides the generation process based on attributes or textual context. These methods allow GANs to generate coherent, diverse, and semantically relevant images in a manner that is fundamental to many contemporary digital art and image creation applications.

#### 4.2.1 Noise-to-image generation

Noise-to-image generation is one of the fundamental applications of Generative Adversarial Networks (GANs), which uses a random noise vector to generate visually coherent digital images by adversarial learning. During training, the generator tries to approximate the distribution of random noise to a correct representation of an image, and the discriminator classifies generated samples as real or fake. In the context of text-to-image synthesis, Generative Adversarial Networks Omar et al. (2023) proposed CapGAN to enhance the feature representation and semantic consistency of the generated images by adding capsule networks into the GAN structure. Their model showed that features extracted based on capsules could improve the preservation of structural information and artistic object composition during capsule generation. Wang et al. (2023) also introduced MemoryGAN, which uses heterogeneous memory modules to help the generator remember the visual patterns of compositions and enhance the quality and diversity of images generated. These methods play a crucial role in the generation of digital works and help to create highly detailed and visually creative images from random latent inputs.

#### 4.2.2 Semantic synthesis

Semantic synthesis is the process of synthesizing images that are not random but rather created based on semantic attributes, labels of objects or context information. This method allows for the generation of images that have coherent themes and useful architectural features. To address this issue, Jin et al. (2023) introduced a gradual multi-granularity semantic fusion framework (GMF-GAN), which fuses multiple semantic representations in the process of text-to-image generation. They progressively merged fine-grained semantic details to bridge the gap between text descriptions and visual outputs in their model. By focusing on key aspects of the context, their approach allowed for greater semantic control when generating images. These studies validate the usefulness of semantic synthesis methods in increasing the interpretability, controllability, and artistic coherence of the digital images generated by GAN.

### 4.3 Style transfer techniques

#### 4.3.1 Artistic style transfer

Artistic style transfer is a technique that uses the visual style of a source image to derive a target image’s brush strokes, texture, and color composition while maintaining the same content structure Wang X. et al. (2025). The GAN-based style transfer method has made it possible to achieve different styles of rendering in art, without the need for human intervention. Habib et al. (2024) addressed the development of GAN architectures for artistic style synthesis, highlighting the high-level artistic abstractions and stylistic semantics that GANs are able to capture. In their survey, they emphasized the increasing importance of oppositional learning in creating paintings, sketches, and other abstract art forms that are human-like in their creativity. To enhance visual consistency in generated artworks, Yuan et al. (2024) proposed RII-GAN, which utilizes multi-scaled aligning strategies and reversed image interaction networks. Their method preserved the fine-grained texture and semantic information of art, which is very appropriate for developed digital art production and creative design use.

#### 4.3.2 Cross-domain translation

Cross-domain translation refers to a process of transforming images from one visual domain to another while retaining their key structural characteristics. The method is often applied when sketching into painting, photo-to-anime, or real-life-to-painting. Liu et al. (2023) showed that conditional GANs with attention mechanisms can achieve semantic domain adaptation and keep the image realistic. Ouyang et al. (2024) then enhanced the fine-grained text-to-image generation task by including contextual linguistic guidance and image translation mechanisms to enable complex artistic translations across visual domains with high accuracy. These cross-domain translation techniques using GAN can be used to facilitate artists and designers in creating and completing creative processes more efficiently and finding new expressions.

### 4.4 Text-to-art generation

Text-to-art generation involves the generation of visual artworks from textual descriptions using a GAN, which allows for direct translation of text to image. It features prompt-based generation, where textual instructions are used to generate images, and language-guided synthesis, which leverages more sophisticated semantic and contextual understanding to create more accurate images. These methods, combined, create higher controllability and creativity in AI-generated imagery, enabling users to generate highly relevant and expressive digital artwork from natural language inputs.

#### 4.4.1 Prompt-based generation

Prompt-based generation helps users to generate digital artwork through a text description, which guides the GAN model in the process of image synthesis. This technique has become a fundamental component of AI-assisted creativity. Omar et al. (2023) demonstrated that CapGAN could well produce the visually coherent artistic images from the textual prompts using the capsule representations for semantic understanding. Moreover, Jin et al. (2023) pointed out the need for semantic fusion at different granularity levels to enhance language-to-art correlation when interpreting textual prompts. These systems enable a user to create a digital painting, fantasy scene, and conceptual artwork from text input alone.

#### 4.4.2 Language-guided synthesis

Language-guided synthesis extends prompt-based generation and offers increased linguistic knowledge and context-awareness during image generation. Ouyang et al. (2024) introduced a fine-grained framework for text-to-image synthesis with the ability to interpret the semantic relationships in textual descriptions. Their method led to better positioning of objects, consistency of attributes, and realism in generated artwork. Yuan et al. (2024) also introduced multi-scale semantic alignment to improve the language-guided synthesis of images, where the generated results align well with the complex language instructions. These approaches can greatly enhance creative agility and enable interactive AI-based art generation systems.

### 4.5 High-resolution art generation

In GANs, high-resolution art generation is heavily focused on creating and refining detailed, high-quality images. It involves progressive GANs, which gradually increase the resolution during training, and super-resolution GANs, which restore fine details from low-resolution images. These techniques can be used to create high-resolution, realistic, and detailed digital images.

#### 4.5.1 Progressive GAN

The Progressive GAN technique produces high-resolution artwork by progressively increasing the resolution of the image during training. This progressive learning approach helps to establish a stable training process of GAN and improves the quality of images. According to Habib et al. (2024), progressive training methods can help GANs learn global structures and fine art textures. Progressive GANs generate high-quality, highly realistic images that can be used in professional digital art and still capture nuances of their visual content, using incremental training of visual details. They can be very helpful for creating high-quality concept art, landscape paintings, and detailed character portraits.

#### 4.5.2 Super-resolution GAN

Super-resolution GANs aim to improve the resolution and clarity of low-resolution images, while maintaining the artistic textures and semantic details. Wang et al. (2025) proposed a new GAN design called SP-IGAN to super-resolve real-world images with semantic prior knowledge. The results were more accurate than the other techniques when applied to high resolution artworks, with their method maintaining more complex texture, edges and the stylistic characteristics.

### 4.6 3D art and scene generation

GANs are utilized for generating 3D art and scenes, which is useful for creating realistic 3D objects and environments. It has 3-D GANs for volumetric modelling and neural rendering for realistic scene synthesis. They are commonly employed in VR, gaming, and digital applications.

#### 4.6.1 GANs

3D GANs generate 3D objects and scenes, rather than images, by learning the volumetric or geometric representation. The models can be used to generate realistic environments, sculptures, avatars, and virtual assets, which are crucial for gaming and metaverse applications. Habib et al. (2024) discussed the increasing relevance of 3D generation techniques based on GAN in digital creativity in an immersive context, such as virtual reality and interactive art systems. By combining semantic conditioning with adversarial learning, structurally consistent and detailed 3D artistic objects can be generated.

#### 4.6.2 Neural rendering

Neural rendering is a combination of deep learning and computer graphics, allowing for the generation of photorealistic scenes by learned representations. Neural rendering with GANs is a computational method that is used to generate complex lighting, textures, and spatial relationships within digital environments. Yuan et al. (2024) showed that multi-scale alignment mechanisms can enhance the realism of scenes and continuity of textures in the rendering process. The use of these techniques is growing in visual effects in movies, virtual production, and AI-generated virtual environments.

### 4.7 Interactive and adaptive GANs

With interactive and adaptive GANs, users can engage dynamically and generate images quickly. They range from human-in-the-loop systems that involve user input to produce customized outputs to real-time GANs that produce images in real-time with minimal delay. Such strategies stimulate creativity, control and responsiveness in interactive digital art and multimedia applications.

#### 4.7.1 Human-in-the-loop systems

More human-in-the-loop (HITL) GAN systems engage with user feedback throughout the generation process, enhancing the personalization and creativity of the image. According to Habib et al. (2024), interactive GAN frameworks enable the artist to tweak the generated output by altering the style, composition, or semantic information in an iterative manner. The goal of these systems is to harmonize the creative process of humans with the capabilities of the machines for a collaborative production of digital art.

#### 4.7.2 Real-time generation

Real-time GAN generation refers to the ability to produce artworks in real time with minimal processing time. Ouyang et al. (2024) pointed out that the efficiency of generating images could be improved by using effective semantic synthesis methods, so as to obtain better results in terms of image generation time and image quality. Real-Time GAN systems have been widely used in live digital painting, interactive multimedia, augmented reality filters, and games where the rendering result is required in real time.

### 4.8 Emotion-aware art generation

Emotion-aware GANs generate an image based on human emotions, such as happiness, sadness, excitement etc. It features emotion-based generation and emotion computing to produce emotion-expressive and adaptive visual outputs.

#### 4.8.1 Sentiment-driven generation

Sentiment-driven generation refers to creating artwork with a certain feeling, like happiness, sadness, excitement, or calmness. GANs process the emotional content of the text, faces, or background context and produce artworks with emotional expressions. Regarding modern GAN architectures, Habib et al. (2024) discussed various aspects of emotionally resonant image generation. Such systems are increasingly being applied in therapeutic art applications, emotional storytelling, and custom digital experiences.

#### 4.8.2 Affective computing

Affective computing for the generation of adaptive artistic content in GANs emphasizes the computational representation and modelling of human emotions. Yuan et al. (2024) showed that using semantic alignment and contextual interaction networks can enhance emotional agreement in generated images. The integration of affective computing with GANs can also enable digital art systems to adapt their responses to users’ emotional states and produce personalized artistic experiences that are more emotional and realistic.

## 5 Application-based classification

Application-based Classification of GANs refers to their various applications in real-world domains like gaming, animation, film, VR and Metaverse, NFT and Crypto Art, and music visualization. The applications illustrate the use of GANs for automated and real content creation in creative and multimedia sectors.

### 5.1 Gaming

In the gaming industry, GANs are extensively used for character design, environment generation, texture synthesis, and procedural content creation. The goal of a GAN is to create a character, costume, terrain or virtual world, without requiring manual design, while saving time and work for the game developer. These architectures enhance creativity by generating a variety of rich and realistic game assets from small training sets. Lataifeh et al. (2023) showed that GANs can help improve the creativity of character designers by producing novel character concepts and character styles for use in games. In a further development of this concept, Lataifeh et al. (2024) suggested a human–machine co-creation model, where GANs assist designers in creating gaming characters with varying visual styles and artistic content. In a similar vein, Hu et al. (2024) presented an interactive GAN-based design generation system for spatial computing applications, which is applicable to optimizing the environment and interactive virtual scene generation in gaming systems. Furthermore, Wang et al. (2023) introduced MemoryGAN, an approach to compositional image synthesis that allows for the creation of more realistic and structurally consistent gaming environments.

### 5.2 Animation

GANs have significant applications in the field of animation such as rendering, synthesis, enhancing movement, and frame interpolation. These models can be used in real-life animation scenes, to make the animation more fluent, and reduce the complexity of the animation process and rendering time. Using GAN-based animation systems can automatically generate background sets, facial expressions, and intervening actions, which helps to streamline the animation production process. In Hu et al. (2024), an interactive and generative method using GAN for generating visually consistent animated environments on spatial computing platforms was designed. Another study found that Zhang and Schomaker (2024) explored how to optimize the latent space of a conditional GAN with a focus on controlled semantic changes and seamless image manipulation to benefit image animation. These methods are essential for a consistent and realistic movement, and for greater flexibility in animation production, which makes it more automated.

### 5.3 Film industry

In the film industry, GANs are extensively used in Visual Effects, Deepfakes, Face Synthesis, Scene Reconstruction, and CGI Enhancement. GANs create photorealistic characters, enhance backgrounds digitally and simulate facial expressions, which enhances the realism of the cinematic experience. The technologies are also being incorporated in movie production pipelines to cut down the cost of editing and enhance the quality of special effects. In a comparative analysis, Al Kishri et al. (2025) examined GAN-based adversarial techniques for manipulating faces, revealing that adversarial models produce highly realistic synthetic faces for creating film and entertainment characters. Likewise, Say et al. (2025) examined the artefacts and detection techniques of deepfakes created by GANs and proposed methods to tackle authenticity issues in film media. Moreover, Deressa et al. (2025) introduced GenConViT, a generative convolutional vision transformer framework for detecting deepfake videos, enhancing the reliability and security of synthetic media content in the film production process. Javed et al. (2025) also proposed a multimodal deepfake detection approach by combining local–global feature learning with diffusion models to detect video manipulations produced by powerful GAN models.

### 5.4 VR and metaverse

GANs are notable in VR and Metaverse applications for generating realistic avatars, virtual assets, creating immersive environments, and modelling interactive worlds. The models can be used to generate dynamic and visually consistent virtual environments, enhancing user immersion and engagement in digital environments. The systems can create personalized avatars, realistic textures, and adaptive virtual objects, all of which can be integrated into metaverse environments, based on the GAN. Lataifeh et al. (2024) demonstrated the use of co-creative approaches using the GANs approach to generate personalized avatars and character models for use in immersive virtual environments. Similarly, Hu et al. (2024) proposed interactive GAN-based spatial computing systems that allow for the generation and optimization of virtual assets in real-time for VR applications. Furthermore, Wang et al. (2023) developed MemoryGAN, a tool for compositional image synthesis, which lays the foundation for producing intricate virtual environments and scene components on metaverse platforms.

### 5.5 NFT and crypto art

The use of GANs has transformed the world of NFT and crypto art, making art production more automated, creating unique digital artworks, and producing visuals by AI. The systems based on GAN can produce new art with unique visual patterns, and they are very suitable for the owners of blockchains and NFT marketplaces. These architectures enable an artist or designer to produce a wide variety of outputs of art with minimal human input. In a recent study, Lataifeh et al. (2023) showed that GANs can be used to enhance artistic creativity by creating novel visual character designs and artistic concepts for digital collectables. Similarly, Zhang and Schomaker (2024) explored interpretable latent space optimization in conditional GANs, which allows for the generation of personalized NFT assets and controlled creation of AI-generated artworks. Furthermore, Hu et al. (2024) introduced interactive GAN-based creative systems capable of producing personalized creative content for digital art platforms and metaverse economies.

### 5.6 Music visualization

GANs are increasingly used in music visualization for generating audio-reactive visual art, synchronized multimedia effects, and immersive artistic experiences. These models are based on the idea of dynamically visualizing the musical patterns, rhythms and semantic information in response to the audio input. Music visualization systems using GAN are used to increase the interaction and participation of the concert audience, digital entertainment, and multimedia art installations. Hu et al. (2024) presented interactive visual generation systems using GANs which can produce music visualization applications with adaptive visual effects and immersive multimedia environments. Also, Zhang and Schomaker (2024) investigated conditional architectures of text-to-image GANs using semantically informative latent representations for semantic and audio-conditioned image generation. These techniques play a crucial role in creating AI-generated audio-reactive visual art and synchronized multimedia interactions within digital entertainment systems.

### 5.7 Dataset-based foundations for GAN training and evaluation

Data sets are crucial to the performance of GAN, both for training and testing. There are standard datasets with different visual and semantic information, and custom datasets for domain-oriented applications. These datasets enhance the quality, realism and controllability of outputs from GANs.

#### 5.7.1 WikiArt dataset

The WikiArt dataset is widely employed in artistic image generation applications, since it has a large number of paintings from various artistic movements and painters. This dataset is used to train GAN models for artistic composition, color blending, texture formation, and painting style. According to Khan et al. (2024), WikiArt allows AI systems to learn and mimic the artistic style of human-made artworks. Their study showed that the art produced by GANs trained with artistic data sets could closely simulate visual aesthetics and be clearly distinguished from human art with structural patterns. Zhang and Schomaker (2024) have pointed out that WikiArt datasets enhance latent space optimization and semantic consistency in conditional text-to-image GAN models.

#### 5.7.2 CelebA dataset

For the face generation and manipulation task of facial attributes, the CelebA dataset is one of the most popular datasets. It includes images of celebrities and their faces labelled for various facial features such as expressions, haircuts, sex and age. Wei and Wang (2024) introduced a parallel GAN framework for facial attribute editing and separation with the CelebA dataset. In their study, they have demonstrated that the data enables the correct manipulation of facial features while preserving the identity of the face and its realism. In addition, Sowmya et al. (2024) used CelebA for attentional multimodal GAN-based face synthesis, demonstrating that the dataset improves attention learning and enhances the generation of realistic human facial images.

#### 5.7.3 ArtBench dataset

ArtBench dataset is designed for artistic image classification and Art Generation Benchmarking. It is comprised of categorized art samples, which are used to teach GAN models various artistic styles and visual semantics. The set is popularly used to test the performance of GAN architectures in digital art generation. Khan et al. (2024) pointed out the necessity of benchmark datasets of artworks to compare AI-generated works with human ones. Their findings showed that artistic benchmark datasets can aid in aesthetic assessment, style analysis, and creative pattern recognition for AI-generated images. Moreover, Zhang T. et al. (2023) showed that transformer-based GAN architectures trained on structured artistic datasets outperform their counterparts in terms of synthesis efficiency and visual quality.

#### 5.7.4 Danbooru dataset

The Danbooru dataset is a huge anime illustration dataset, which is widely utilized for synthesizing anime images or generating anime characters. It includes millions of annotated anime images based on facial expression, pose, costume and artistic style. The dataset is used to train GAN models to capture the visual patterns and semantic relationships of anime. Zhang and Schomaker (2024) explained that datasets containing rich semantic tags, which enhance controllable image generation and latent space interpretation in conditional GAN models. Semantic annotations are highly relevant to the GAN application on Danbooru-based images, as they enable ANIME image synthesis and attribute control.

#### 5.7.5 ImageNet dataset

The ImageNet dataset is a very popular dataset in GAN research due to its millions of images with thousands of categories labelled. The dataset offers a broad understanding of visual information, which can be used to enhance feature learning, transfer learning, and generate large-scale synthetic images. To overcome these challenges, Zhang et al. (2023) used large-scale image datasets to enhance the performance of transformer-based GAN for fast and efficient image synthesis. They found that large and diverse datasets enable the GAN models to be more scalable, with better image quality and better semantic representation learning. ImageNet is also commonly used for pretraining the GAN encoders and stabilizing the adversarial learning process.

#### 5.7.6 Custom datasets

Researchers create custom datasets for domain-specific GAN tasks like medical imaging, creating virtual avatars, synthesizing cultural artwork, or personalized digital art. The datasets have been compiled based on the application requirements and can contain manually annotated samples. Khan et al. (2024) used a set of custom-trained AI-generated and human-made images to examine the relationship between machine-generated and human-created art by analyzing similarities and differences in their styles. They showed that custom datasets offer more specific evaluation and optimization of GAN models for application. Likewise, Wei and Sowmya et al. (2024) adapted subsets of images and employed tuning of preprocessing methods for the CelebA dataset to enhance the editing performance and realistic face synthesis results of the facial attributes.

## 6 Aesthetic evaluation of GAN-generated art

The aesthetic evaluation of GAN-generated art assesses the ability of AI to create realistic and artistic art that closely mimics human creativity. It brings together objective measurement with subjective human evaluation and focuses on creativity, interpretation and fair assessment of created works.

### 6.1 Importance of aesthetic evaluation

The aesthetic evaluation of GAN-generated art is crucial for assessing the effectiveness of AI in capturing human ideas about creativity, realism, emotional expression, and artistic value. Artistic evaluation is subjective in nature and not like conventional image analysis, as the viewer interprets beauty and meaning differently depending on the cultural background, personal experience and contextual interpretation. Meng et al. (2023) pointed out that the aesthetic perception of dynamic generative art can change with the passage of time, and it relies on visual and temporal aspects when evaluating art rather than just visual aspects. Similarly, Stein et al. (2023) pointed out that traditional GAN evaluation metrics can be biased and favour specific GAN architectures or datasets, potentially resulting in misjudgements of artistic creativity and realism. Furthermore, Yang et al. (2023) indicated that realism, diversity, and semantic consistency are hard to measure by a single metric and, therefore, multidimensional evaluation frameworks are needed for full evaluation. Moreover, Zhang and Schomaker (2024) showed that the degree of interpretability of the latent space is crucial for the controllability and perceived quality of AI-generated art, particularly in the context of text-to-image models, where aligning user inputs with artistic outputs remains a challenge.

### 6.2 Objective evaluation metrics

Objectives evaluation measures image quality, realism and similarity between generated and real images quantitatively. These metrics are widely used because they offer repeatable and automatic evaluation processes.

#### 6.2.1 Inception Score (IS)

Inception Score (IS) is used to assess the quality and diversity of images produced by GANs by passing images through a pretrained neural network. It measures the confidence level of the classification of images and the diversity of the samples produced by classes. But, IS does not account for semantic correctness or artistic creativity, and sometimes even provides misleadingly high scores for images that are not realistic. (Yang et al., 2023).

#### 6.2.2 Fréchet Inception Distance (FID)

Fréchet Inception Distance (FID) is a popular method for assessing GANs that measures the statistical distance between the distribution of features of real images and generated images. It quantifies the similarity of the generated images to real images, with a lower value representing better quality and similarity. FID is sensitive to feature extraction methods, dataset size and preprocessing steps, which can impact reproducibility (Duminil et al., 2024). In the same vein, Stein et al. (2023) pointed out that even very good perceptual quality does not necessarily lead to FID not penalizing certain models.

#### 6.2.3 Peak Signal-to-Noise Ratio (PSNR)

Peak Signal-to-Noise Ratio (PSNR) is a ratio used to evaluate the quality of the reconstruction by computing the difference between the reconstructed image and the original image at each pixel. It is often applied for assessing the quality of the reconstructed image, particularly in super-resolution applications. Wang M. et al. (2025) found that the higher the PSNR value, the better the structural reconstruction, but it does not necessarily represent the higher perceptual or visual quality.

#### 6.2.4 Structural Similarity Index Measure (SSIM)

Structural Similarity Index Measure (SSIM) is an image quality evaluation based on luminance, contrast, and structural information of the generated image with reference to the reference image. This is broadly considered to be a measure of the ability of GANs to maintain visual structure and texture. As noted in Park et al. (2025), SSIM works well for assessing structural consistency for the high-resolution outputs, but may not be sufficient for evaluating artistic abstraction and stylistic originality.

### 6.3 Subjective evaluation and media psychology perspectives

Subjective evaluation of GAN-generated art goes beyond traditional studies of human perception, focusing instead on the psychological processes involved in aesthetic perception, emotional reaction, and cognitive processing. Objective metrics such as FID and IS measure technical aspects, while subjective evaluation reflects the phenomenological aspects of the audience’s perception, interpretation, and emotional response to the AI-generated artworks. In their studies, Meng et al. (2023) showed that the audience’s response to the works of art created by GAN evolved over time, which indicates that the perception of beauty is situational and is a dynamic process. Gajewska (2025) noted that the level of sophistication that AI can attain in creating art is not easily distinguishable from human-created art, and therefore, there are profound questions about aesthetic judgment. He et al. (2025) demonstrated that the colors, semantic consistency, and prompt alignment play significant roles in the satisfaction of viewers and their artistic preferences. But the psychological effect of GAN-generated art can only be understood by analyzing the intersections between media psychology paradigms, such as cognitive load, processing fluency, emotional valence, and audience-centered perception paradigms, and subjective aesthetic evaluation.

#### 6.3.1 Cognitive load in aesthetic perception

The theory of cognitive load is a theory from educational psychology that can be used to gain insight into the way in which viewers process GAN-generated artworks. Cognitive load is a measure of the total amount of working memory resources that are engaged in the processing of information. For AI-generated art, cognitive load can be affected by several factors: visual complexity, semantic ambiguity, stylistic novelty, and how far the piece deviates from the norms of art.

Viewers see GAN-generated images and are required to perform a dual processing task: Perceptual processing of visual features (color, texture, composition) and semantic processing of meaning, intent, and artistic value. High complexity GAN outputs could include more intricate details, surreal elements, or hybrid styles which may require more cognitive effort, either intensifying engagement (if the complexity is engaging and rewarding to the viewer) or causing cognitive overload (if the image is too complex to be processed).

On the other hand, overly formulaic or simplistic GAN outputs can lead to low cognitive load, yet low aesthetic interest. Aesthetic appreciation has an inverted-U relationship with the optimal cognitive load, with moderate complexity providing the most engagement and pleasure. Balanced images, neither too chaotic nor too predictable, are more aesthetically pleasing in the eyes of viewers when produced by GAN architectures. This is consistent with the arousal theory in media psychology that suggests that the aesthetic preference is at its highest when the stimulus is of intermediate complexity and thus causes moderate arousal. In the design of GAN systems for entertainment applications (such as games, animation, interactive media), it is important to carefully tailor the level of cognitive load to the target user’s expectations and processing power. For example, in a casual game environment, it may be preferable to have assets produced by a simpler GAN that would not overload the player’s perceptual system, whereas in an art application, it might be desirable to produce a higher complexity image.

#### 6.3.2 Processing fluency and aesthetic preference

Processing fluency is important to aesthetic judgment because it is the subjective ease with which information is processed. Processing fluency theory of aesthetic pleasure states that stimuli that are easier to process are generally preferred, all else equal. Visual clarity, symmetry, prototypicality, semantic coherence are factors that affect fluency. Processing fluency can differ widely between different architectures and training protocols in the context of GAN-generated art. Images generated by StyleGAN models tend to have high processing fluency, which results in higher aesthetic preference for general audiences; images tend to be structurally coherent and have high fidelity to the real world.

On the other hand, if the early GAN architectures or models are trained on small datasets, they can produce images that contain artifacts, inconsistencies, or surreal distortions, which can hinder the fluency of the processing and even lower the aesthetic value. But there is no direct correlation between fluency and preference for aesthetics. Moderate disfluency can be advantageous in certain artistic contexts (abstract art, surrealism, experimental media) to generate curiosity, surprise, or further cognitive involvement, while high fluency, in general, would improve preference for realistic or representational art. The greater the versatility of the GAN system to be able to modulate processing fluency, the more output can be very fluent (photorealistic) and very strategically disfluent (abstract, surreal), the more versatility there is in art. Empirical research on media psychology indicates that processing fluency is related to viewer experience and expectations. The general public might favor the high-frequency GAN outputs, whereas art aficionados may appreciate the disfluent or unusual GAN-generated artworks that defy perceptual norms. This suggests that the subjective aesthetic quality assessment of GANs should consider the segmentation of the audience and contextual factors.

#### 6.3.3 Emotional valence and affective response tracking

Aesthetic experience has a basic dimension, namely emotional valence, in terms of how positively or negatively a stimulus is experienced. Depending on the visual content, stylistic choices and context framing, GAN-generated art can evoke a wide range of feelings, from happiness and wonder to discomfort and sadness.

As mentioned in Section 4.8, emotion-aware GANs explicitly integrate affective computing to produce emotional images. But even traditional GANs are subtly shaping emotional valence by producing color palettes, facial expressions, environmental atmospheres, and symbolic imagery. For example, portraits created by GANs that are warm in colour and have soft lighting tend to induce positive emotional responses, whereas portraits with darker colour palettes, distorted faces, or threatening images can induce negative emotional responses.

Multimodal assessment approaches are needed to track emotional valence in response to GAN-generated art. Traditional self-report methods (Likert scales, semantic differentials) allow for explicit ratings of emotional experience and can be influenced by social desirability bias and by introspective access. Physiological measures (HRV, skin conductance, facial EMG) provide objective measures of emotional arousal, but are not specific to valence. New models integrate self-reported, behavioral, and physiological responses to AI creations to create detailed affective profiles of viewers’ reactions.

Emotional valence is related to other psychological factors from a media psychology point of view, including empathy, presence, and narrative engagement. Emotional resonance is a quality of interactive media (video games, virtual reality) characters that GAN generates successfully, which can increase the user’s immersion and attachment. On the other hand, negative emotions (uncanny valley effects in synthetic faces) can have a detrimental effect on the user experience and decrease the acceptance of AI-generated content.

#### 6.3.4 Media psychology paradigms in AI art evaluation

Several key paradigms in media psychology are relevant to subjective aesthetic evaluation of GAN-generated art: According to the Uses and Gratifications Theory, audiences actively seek out media content to satisfy certain psychological desires, including entertainment, aesthetic enjoyment, social bonding, and self-expression. The technical quality of GAN-generated art is not the only criterion by which it is to be judged in digital entertainment contexts, but also its ability to meet the various user motivations.

A GAN-generated game asset can be technically amazing, but not capture the user’s attention if it’s not in line with their gratification-seeking behaviors. The Parasocial Interaction Theory, which was first used in the context of media relationships, can be applied to AI-generated characters and avatars. In games, movies, or virtual worlds, viewers might develop parasocial relationships with the GAN-generated characters, which can shape their tastes and feelings. Assessing the generated characters’ ability to induce parasocial interaction is a crucial part of evaluating GAN-generated characters, in addition to their visual realism.

The Parasocial Interaction Theory, which was first used in media relations, can be applied to AI-generated characters and avatars. In gaming, film, or virtual worlds, viewers can develop parasocial relationships with the characters created by the GAN, which can shape their preferences and emotional attachment to the content. Assessing the quality of the generated characters from the GAN must be done not only on the basis of realism but also on the basis of their ability to provoke parasocial interaction.

The Flow Theory, a psychological phenomenon of being totally absorbed in an activity, is very applicable to interactive GAN applications. For real-time content generation (procedural game environments, adaptive music visualization) GAN systems need to ensure optimal challenge-skill balance to enable flow. Such systems should be evaluated both on their aesthetic appeal and their ability to enhance user flow and engagement. Although the Uncanny Valley Hypothesis was first introduced to robotics, it has far-reaching implications for the human faces and avatars created by GANs. When GAN’s output is close to photorealism, the aesthetic and emotional response to the image may be reduced when the image is almost but not quite human. The evaluation of GAN-generated faces must be done with great care and sensitivity to the uncanny valley effects, especially in the fields of synthetic humans (deepfakes, virtual influencers, digital avatars).

The incorporation of these media psychology concepts into subjective evaluation systems allows for a more comprehensive analysis of GAN-generated artwork that considers various factors, such as technical aspects, psychological processing, emotional reactions, and contextual significance. Further studies are needed to create standardised tools that integrate cognitive, affective, and behavioural components to assess the subjective effects of AI-generated artworks in various entertainment and creative contexts.

### 6.4 Creativity assessment

Creativity Assessment is important when it comes to GAN-generated art because it tests the capability of AI systems to create creatively innovative, original, and emotionally engaging artworks. Zhang and Schomaker (2024) have clarified that novelty in AI-generated art relies on the capacity to produce compositional novelty in the latent space representations while maintaining consistency in their meaning. Advanced dual conditional GAN architectures equipped with attention modules that minimize repetitive generation patterns and boost semantic diversity are highlighted by Liu et al. (2023) as a way to enhance originality. Furthermore, Gajewska (2025) noted that emotional reactions are a crucial factor in measuring creativity for viewers as they tend to react more emotionally to works of art generated by AI when the images have expressive visual elements, a coherent composition, and artistic depth comparable to that of human-made paintings.

### 6.5 Explainability and interpretability

Explainability and interpretability are crucial aspects of GAN-generated art, as they enhance the trust, transparency, and understanding of how AI models produce art. In a study by Zhang and Schomaker (2024), they demonstrated that interpretable latent space manipulation can make users grasp the relationship between semantic features and artistic attributes of the resulting artwork, thereby enhancing the trustworthiness and controllability of AI art systems. To prevent false claims from generative models being the superior approach and to provide a fair comparison of generative model architectures, transparency of evaluation protocols was emphasized by Stein et al. (2023). Moreover, Duminil et al. (2024) emphasized the need for standardized benchmarking systems to ensure fair, transparent, and interpretable evaluation of AI-generated images, facilitating responsible and ethical usage of generative art technologies.

### 6.6 Comparative analysis of evaluation approaches

Different metrics for evaluating GAN-generated art are summarized in Table 2, including objective metrics (IS, FID, PSNR, SSIM), human evaluation, expert evaluation, user evaluation, creativity and explainability metrics. It provides evidence that quantitative and qualitative tools need to be used together to provide an overall evaluation of GAN products.

**Table 2:** Comparative analysis of evaluation approaches for GAN-generated art.

Evaluation approach	Method	Advantages	Limitations	Supporting authors
Inception score (IS)	Measures image diversity and class confidence	Simple and widely adopted	Ignores semantic realism and creativity	Yang et al. (2023)
Fréchet inception distance (FID)	Compares feature distributions of real and generated images	Strong correlation with perceptual realism	Sensitive to preprocessing and implementation	Duminil et al. (2024); Stein et al. (2023)
PSNR	Pixel-level reconstruction accuracy	Effective for super-resolution tasks	Poor indicator of perceptual aesthetics	Wang et al. (2025)
SSIM	Structural similarity measurement	Captures texture and structural consistency	Cannot measure artistic originality	Park et al. (2025)
Human perception studies	Viewer-based aesthetic judgment	Captures emotional and subjective quality	Time-consuming and inconsistent	Meng et al. (2023)
Expert evaluation	Assessment by artists and critics	High artistic reliability	Expensive and subjective	Gajewska (2025)
User preference analysis	Survey and ranking-based evaluation	Reflects public acceptance	Influenced by demographic variation	He et al. (2025)
Creativity assessment	Measures novelty and originality	Evaluates artistic innovation	Difficult to standardize	Zhang and Schomaker (2024); Liu et al. (2023)
Explainability analysis	Interprets latent space and generation process	Improves trust and transparency	Complex implementation	Zhang and Schomaker (2024)

### 6.7 Comparative analysis: GANs vs. diffusion models vs. flow matching

The shift from 2024 to 2026 has seen a shift towards diffusion models and flow matching architectures, which have been the front runners of the generative AI in digital art space. This section offers a critical analysis of these emerging paradigms and explains why the industry has slowly moved away from GAN based approaches, and places GANs in the wider context of modern-day generative AI (Table 3).

**Table 3:** Comparative analysis of existing GAN-based studies across architectures, datasets, and applications.

Author	Year	GAN model/framework	Dataset	Application	Metrics	Advantages	Limitations
Al Kishri et al.	2025	GAN-based deepfake models	Deepfake facial datasets	Deepfake facial manipulation analysis	Accuracy, F1-score	Comparative understanding of manipulation techniques	Sensitive to unseen attacks
Crowson et al.	2024	Hourglass diffusion transformer	Large-scale image datasets	High-resolution image synthesis	FID, IS	Scalable high-resolution synthesis	High training cost
Deressa et al.	2025	GenConViT	Deepfake video datasets	Deepfake video detection	Accuracy, Recall	Transformer-enhanced feature learning	Requires extensive data
Duminil et al.	2024	Fidelity evaluation framework	CGI datasets	Fidelity quantification	FID, LPIPS	Comprehensive evaluation study	Metric inconsistencies
Gajewska	2025	AI art evaluation framework	AI-generated art datasets	Human recognition of AI art	Human survey metrics	Cognitive perspective on AI art	Subjective evaluation
Danyi	2025	Contrastive alignment and structural guidance GAN	Text-image datasets	High-fidelity text-to-image generation	FID, CLIP score, Structural similarity	Stable convergence at scale; improved structural guidance	Complex architecture; may require extensive fine-tuning for niche artistic styles
Habib et al.	2024	GAN survey models	Text-to-image datasets	GAN evolution survey	Comparative analysis	Broad review of T2I synthesis	Limited experimental validation
Hatamizadeh et al.	2024	DiffiT	Vision datasets	Diffusion image generation	FID, IS	Transformer-based generation quality	Resource intensive
He et al.	2024	Introspective GAN	Benchmark image datasets	Incremental generation/classification	Accuracy, FID	Dual generation and classification	Training complexity
He et al.	2025	Prompt-guided aesthetic model	Aesthetic benchmark datasets	Image color aesthetics assessment	Accuracy, Correlation	Prompt-aware aesthetic evaluation	Dataset dependency
Hu et al.	2019	Variational conditional GAN	Fine-grained image datasets	Controllable image generation	IS, FID	Fine-grained controllability	Mode collapse risk
Hu et al.	2024	Interactive GAN design system	Spatial design datasets	Design optimization	User evaluation	Interactive co-design support	Limited scalability
Javed et al.	2025	Diffusion + deepfake detector	Multimodal datasets	Deepfake detection	Accuracy, Precision	Local–global feature integration	Computationally expensive
Jin et al.	2023	GMF-GAN	Text-image datasets	Text-to-image synthesis	FID, IS	Multi-granularity semantic fusion	Semantic mismatch issues
Kang et al.	2023	Scaled GAN	Large text-image datasets	Large-scale T2I synthesis	FID	Improved scalability	High GPU requirements
Kas et al.	2024	EigenGAN	Conditional image datasets	Image generation	FID, IS	SVD-based latent representation	Complex optimization
Khan et al.	2024	AI art neural constellation	Human and AI art datasets	AI art comparison	Similarity metrics	Collective analysis of AI art	Interpretability challenges
Lataifeh et al.	2024	GAN co-creation system	Character design datasets	Human-machine creative design	User satisfaction	Supports collaborative creativity	Artistic subjectivity
Lataifeh et al.	2023	GAN creativity augmentation	Character datasets	Designer creativity enhancement	User evaluation	Enhances ideation process	Limited automation
Liu et al.	2023	Dual conditional GAN	Semantic datasets	Semantic synthesis	FID	External attention improves detail	Large memory usage
Lu	2024	USE-CMHSA-GAN	Anime datasets	Anime image generation	FID, IS	Improved anime realism	Limited diversity
Ma et al.	2025	Time media GAN	Interactive art datasets	Interactive art creation	User engagement	Supports dynamic art creation	Evaluation subjectivity
Meng et al.	2023	Statistical aesthetic model	Dynamic artwork datasets	Aesthetic prediction	Correlation coefficient	Time-dependent analysis	Limited generalization
Nigam et al.	2025	Frequency distributed CycleGAN	Image translation datasets	Image translation	FID, SSIM	Frequency-aware translation	Training instability
Omar et al.	2023	CapGAN	Text-image datasets	Text-to-image synthesis	IS, FID	Capsule-based semantic learning	Increased complexity
Ouyang et al.	2024	Fine-grained T2I GAN	Fine-grained datasets	Text-to-image synthesis	FID	Detailed synthesis	Sensitive to prompts
Pal et al.	2024	Artistic GAN Analysis	Art datasets	Training impact analysis	FID, Human evaluation	Explores dataset influence	Limited datasets
Park et al.	2025	NeXtSRGAN	Super-resolution datasets	Image super-resolution	PSNR, SSIM	Improved realism	Large training demand
Rosenberg et al.	2015	Conditional GAN (cGAN)	Benchmark image datasets	Controllable image synthesis, conditional generation	Accuracy, Visual quality assessment	Introduced conditional and controllable generation to the GAN framework	Lower resolution and realism compared to modern architectures; prone to early mode collapse
Sauer et al.	2023	StyleGAN-T	Large text-image datasets	Fast T2I synthesis	FID	Fast and scalable	Requires extensive resources
Sauer et al.	2022	StyleGAN-XL	Diverse image datasets	Large-scale image generation	FID	Handles diverse datasets	High memory consumption
Say et al.	2025	GAN deepfake detection	Mixed deepfake datasets	Deepfake detection	Accuracy, F1-score	Robust artifact analysis	Dataset bias
Seo et al.	2024	Stabilized GAN	Benchmark datasets	Stable GAN training	FID	Reduced instability	Complex preprocessing
Shang et al.	2024	Evolutionary GAN	Benchmark datasets	Evolutionary image generation	FID	Covariance crossover improves diversity	Slow convergence
Shatri et al.	2024	CycleWGAN/ProGAN/DCGAN	Handwritten music datasets	Music image synthesis	Accuracy, FID	Comprehensive GAN evaluation	Domain constrained
Sowmya et al.	2024	Attentional multimodal GAN	Human face datasets	Face synthesis	IS, FID	Multimodal attention improves realism	High training cost
Stein et al.	2023	Evaluation metric analysis	Synthetic image datasets	Metric fairness analysis	FID, Precision/Recall	Reveals metric flaws	No unified metric
Tang et al.	2025	Cross-attention GAN	Person image datasets	Person image synthesis	FID, SSIM	Multi-scale attention improves fidelity	Complex architecture
Tian et al.	2024	Enhanced GAN	Benchmark datasets	Image generation	FID, IS	Improved generation quality	Limited novelty
Vela et al.	2023	Cyclic GAN methods	Art datasets	Artistic image generation	FID	Better image quality	Computational overhead
Wang M. et al.	2025	SP-IGAN	Super-resolution datasets	Real-world super-resolution	PSNR, SSIM	Semantic prior integration	Training complexity
Wang et al.	2024	SCGAN	Distributed datasets	Distributed image generation	FID	Semi-centralized coordination	Communication overhead
Wang et al.	2026	CAFE-GAN	CLIP-based datasets	Attention-aware generation	FID, CLIP score	Multi-scale discrimination	Emerging framework limitations
Wang et al.	2023	MemoryGAN	Compositional datasets	Compositional synthesis	FID	Memory-enhanced generation	Scalability concerns
Wang, X. et al.	2025	Domain generalizable portrait style transfer GAN	Portrait / Artistic datasets	Artistic style transfer, portrait rendering	FID, Style consistency metrics	Achieves different styles of rendering without human intervention; domain generalizable	May struggle with highly abstract or non-portrait artistic domains
Wei and Wang	2024	Parallel GAN	Facial attribute datasets	Attribute editing	Accuracy	Effective attribute separation	Artifact generation
Wiem and Ali	2024	FFGAN	Face datasets	Frontal face generation	FID	Contrastive disentanglement	Limited pose diversity
Xiang	2023	GAN artistic styles	Artistic image datasets	Artistic style generation	Human evaluation	Explores generated styles	Subjective outcomes
Xin	2023	Improved DCGAN	High-resolution datasets	Fast image generation	FID	Fast generation speed	Reduced diversity
Xu et al.	2018	AttnGAN	CUB, COCO	Fine-grained T2I synthesis	IS, R-precision	Attention-driven text alignment	Complex training
Yang et al.	2023	GAN evaluation framework	Synthetic datasets	Image synthesis evaluation	FID, Precision	Revisits evaluation reliability	Metric limitations
Yildiz et al.	2024	Self-attention DenseNet GAN	Single-image datasets	Single-image generation	FID	Self-attention feature extraction	Limited scalability
Yu et al.	2024	AGD-GAN	Ancient mural datasets	Mural sketch extraction	IoU, SSIM	Depth-guided extraction	Heritage-specific
Yuan et al.	2024	RII-GAN	Text-image datasets	Text-to-image synthesis	FID	Multi-scale alignment	Complex architecture
Zhang and Schomaker	2024	Conditional T2I GAN	Text-image datasets	Latent space optimization	FID	Improved interpretability	Latent complexity
Zhang T. et al.	2023	Aesthetic GAN	Photographic datasets	Aesthetic image generation	Aesthetic score	Improved visual aesthetics	Subjective evaluation
Zhang T. et al.	2023	PFGAN	Benchmark datasets	Fast image synthesis	FID	Transformer acceleration	Reduced fine details
Zhang J. et al.	2025	Ultra-high-resolution synthesis framework (flow matching/generative)	Ultra-high-resolution image datasets	High-fidelity art generation, ultra-high-resolution synthesis	FID, Resolution metrics, Perceptual quality	Combines training stability with high inference efficiency for high-fidelity art	Emerging framework; requires significant computational resources for ultra-high resolutions
Zhao	2025	CycleGAN	Visual communication datasets	Style transfer	SSIM, User evaluation	Effective artistic transfer	Content distortion
Li et al.	2024	SwinV2-Imagen	Text-image datasets	Hierarchical text-to-image generation	FID, IS	Transformer hierarchy improves semantic quality and coherence	High computation; requires large-scale training data
Ke et al.	2025	Variational GAN with dual-function discriminator	Semantic datasets	Semantic image synthesis	mIoU, FID	Better semantic consistency via dual-function discriminator	Heavy architecture; increased computational overhead

#### 6.7.1 Diffusion models in artistic applications

Unlike GANs, diffusion models work on a completely different principle, with them learning to denoise a gradual noising process, gradually trying to create a high-quality image from random noise. In artistic creation, recent progress in latent diffusion models has greatly improved the semantic alignment and aesthetic fidelity, making them capable of generating high-quality images that capture multiple concepts in a single prompt, which was often difficult for early GANs (Wang X. et al., 2025). But the denoising process is iterative and needs several forward passes, which makes the inference time higher and more computational overhead than single-pass GAN generation.

#### 6.7.2 Flow matching: the emerging paradigm

Rectified Flow architectures are a newer form of generative modeling, known as flow matching models. Unlike diffusion models, these models learn a continuous transport map that directly maps a simple prior distribution to the target data distribution without an iterative denoising step. Flow matching is a promising solution that strikes a balance between the stability of diffusion models and the efficiency of GANs, making it a viable alternative for generating high-fidelity art (Zhang J. et al., 2025).

#### 6.7.3 Critical analysis: why the industry shifted from GANs

The switch from GAN-based to diffusion and flow-based text-to-image generation can be attributed to multiple factors. First, training stability: GANs are well known to suffer from mode collapse and have to balance generator and discriminator losses, while diffusion and flow models optimize a single well-defined objective function, where the convergence is stable at scale (Dhariwal and Nichol, 2021). Second, semantic controllability: Conditional GANs have been plagued by this issue, and modern diffusion models have shown to be very good at understanding complex textual prompts and producing images that adhere to nuanced stylistic instructions.

#### 6.7.4 Positioning GANs in the contemporary generative AI landscape

In certain areas of digital art and entertainment, GANs still have unique and irreplaceable advantages, even in the face of the emergence of diffusion and flow models. Perhaps most importantly, GANs are capable of real-time generation, as their inference requires only one forward pass, which is suitable for video games and live digital art installations that need to generate content in real time, as well as for interactive media where latency is critical. In this context, recent studies have called for hybrid architectures that judiciously use GANs for interactive real-time generation and diffusion/flow models for offline high-fidelity refinement (Hu et al., 2026). In addition, new psychological models need to be developed to assess the aesthetic value and cognitive effects of these various models in the context of contemporary generative AI environments (Zhang C. et al., 2025). To conclude, the future of digital art AI is not defined by any single architecture but by the thoughtful combination of GANs, diffusion, and flow-based methods depending on the computational limitations and user experience objectives.

## 7 Comparative analysis of existing studies

**Figure 1:**
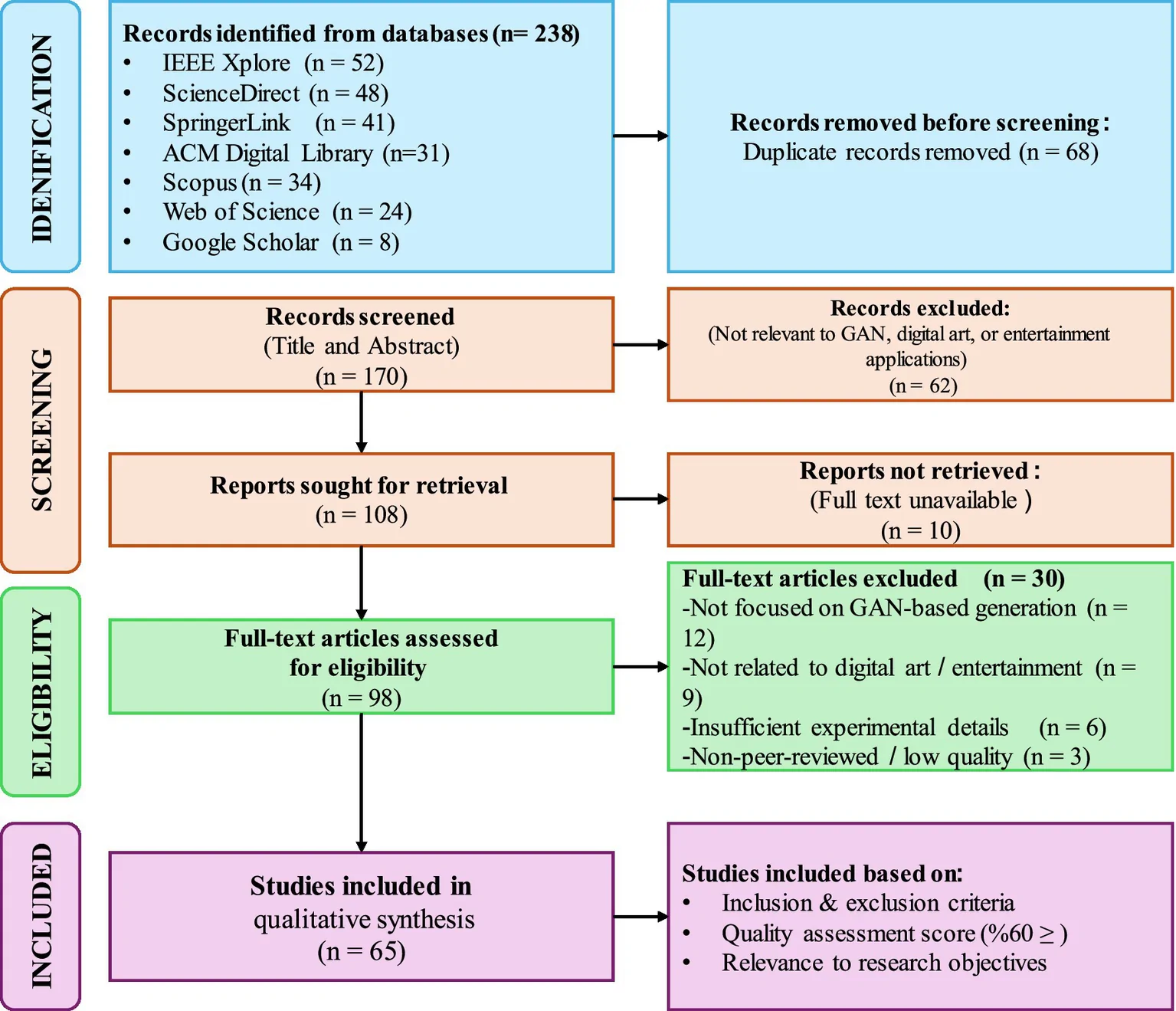
PRISMA flow diagram.

**Figure 2:**
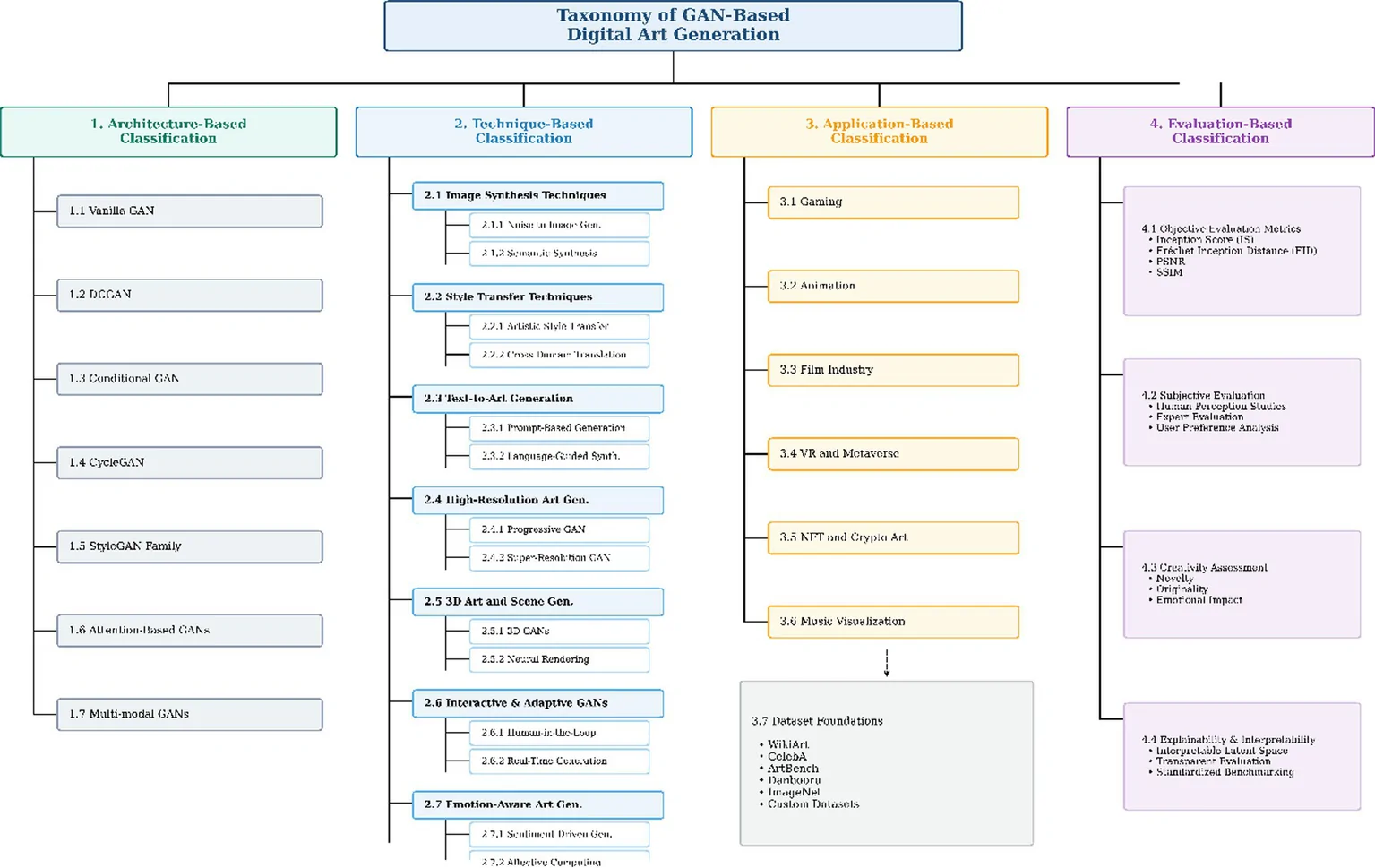
Taxonomy of GAN-based digital art generation.

**Figure 3:**
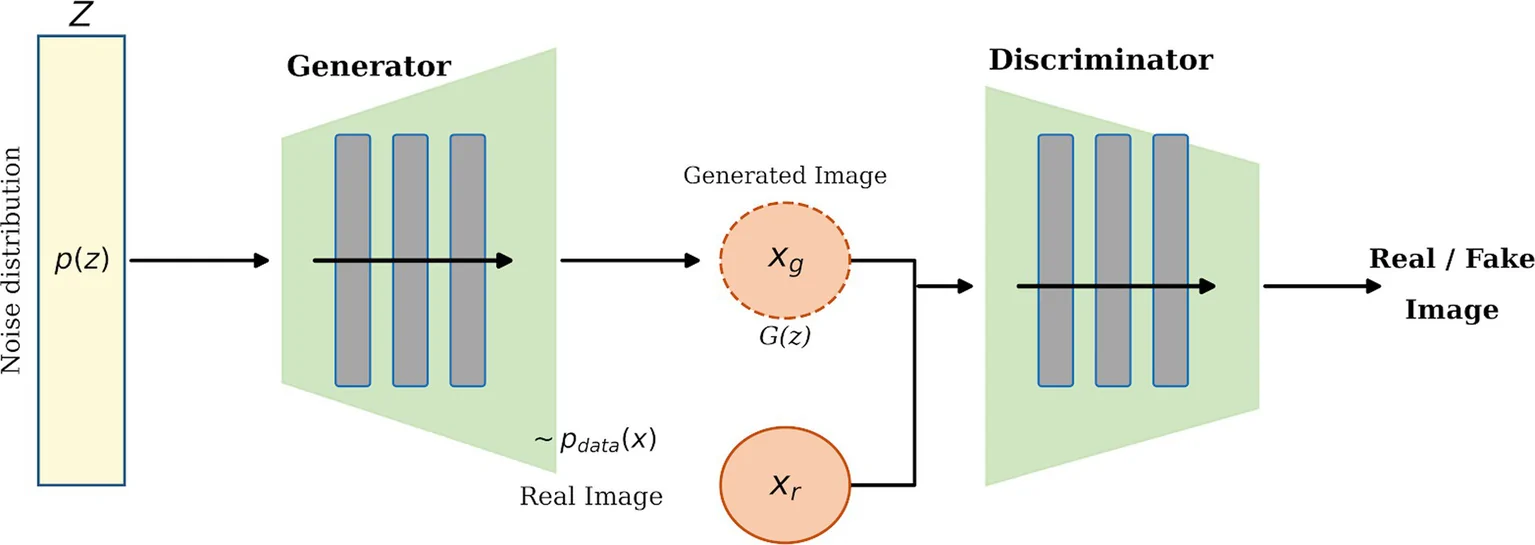
Vanilla GAN.

**Figure 4:**
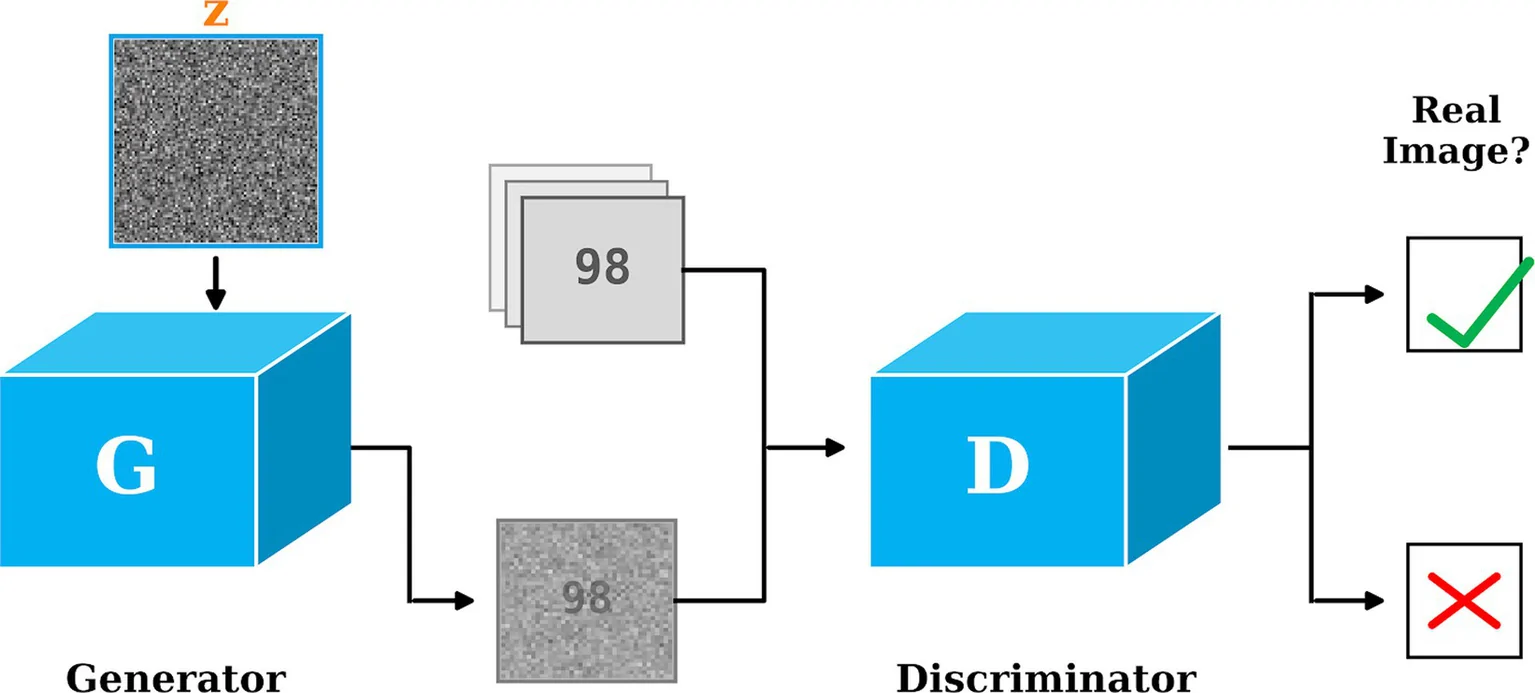
Deep convolutional generative adversarial network.

**Figure 5:**
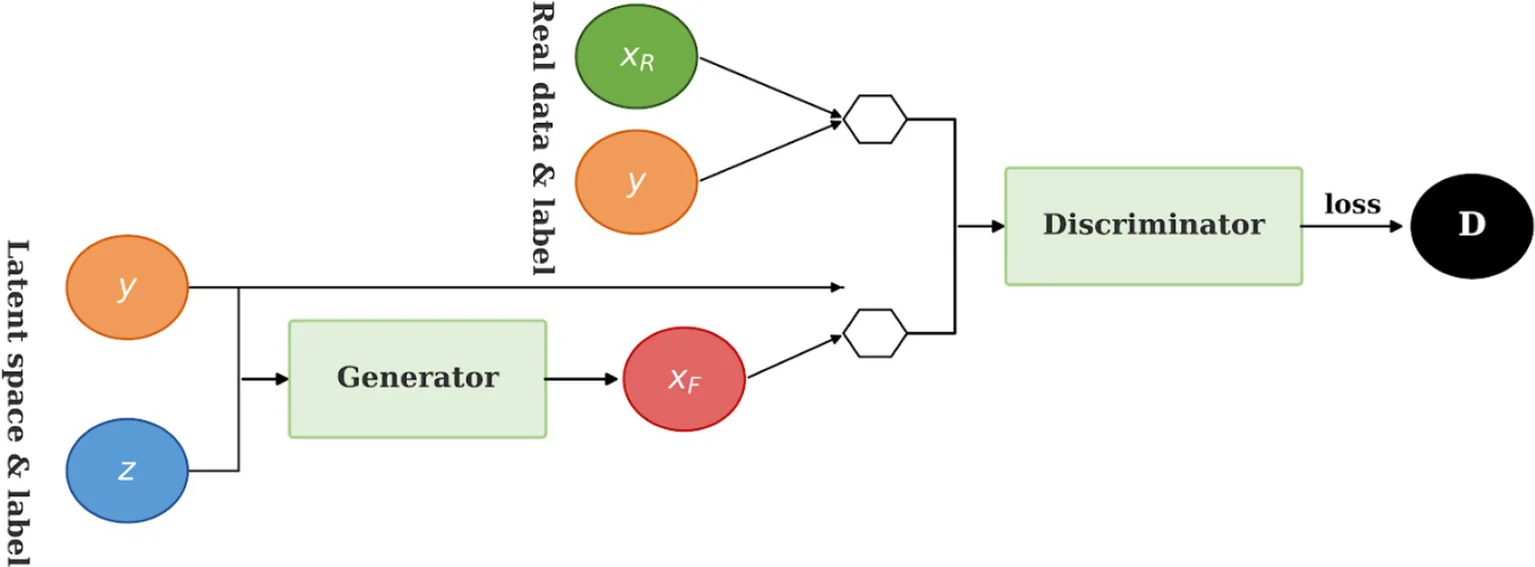
Conditional generative adversarial networks.

**Figure 6:**
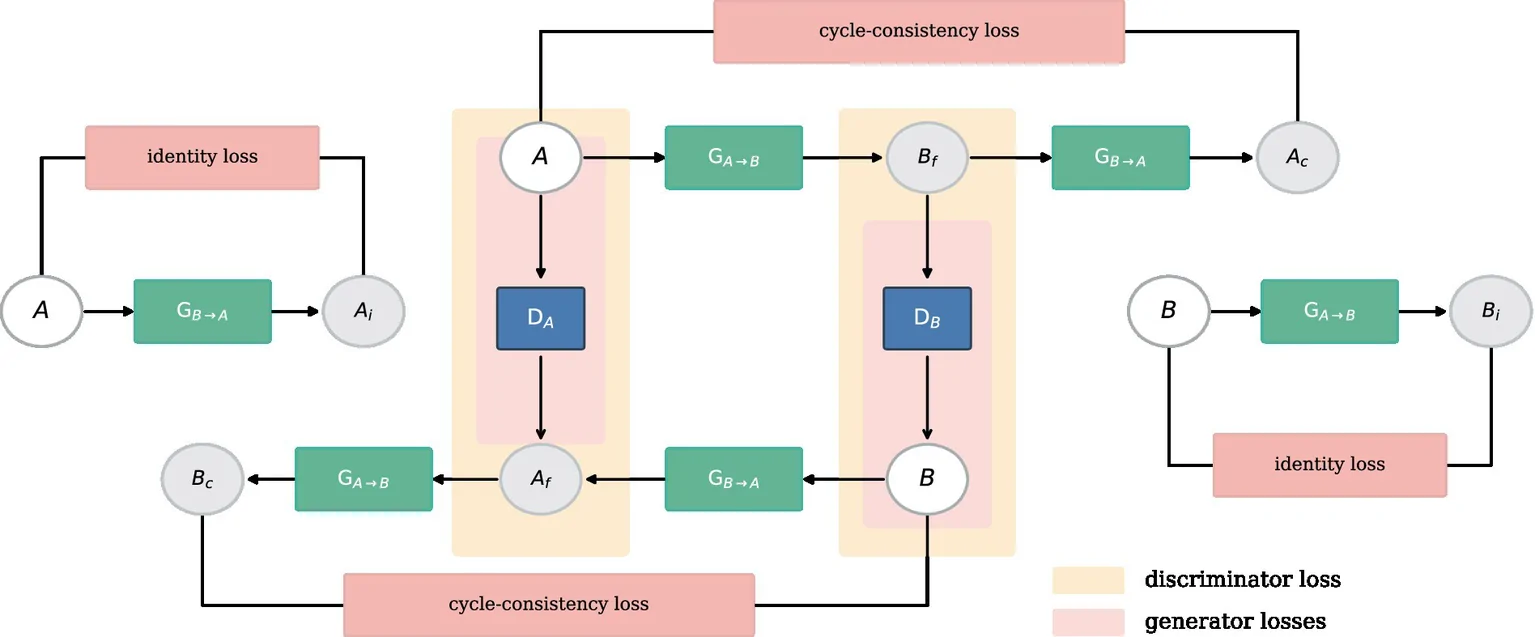
CycleGAN.

**Figure 7:**
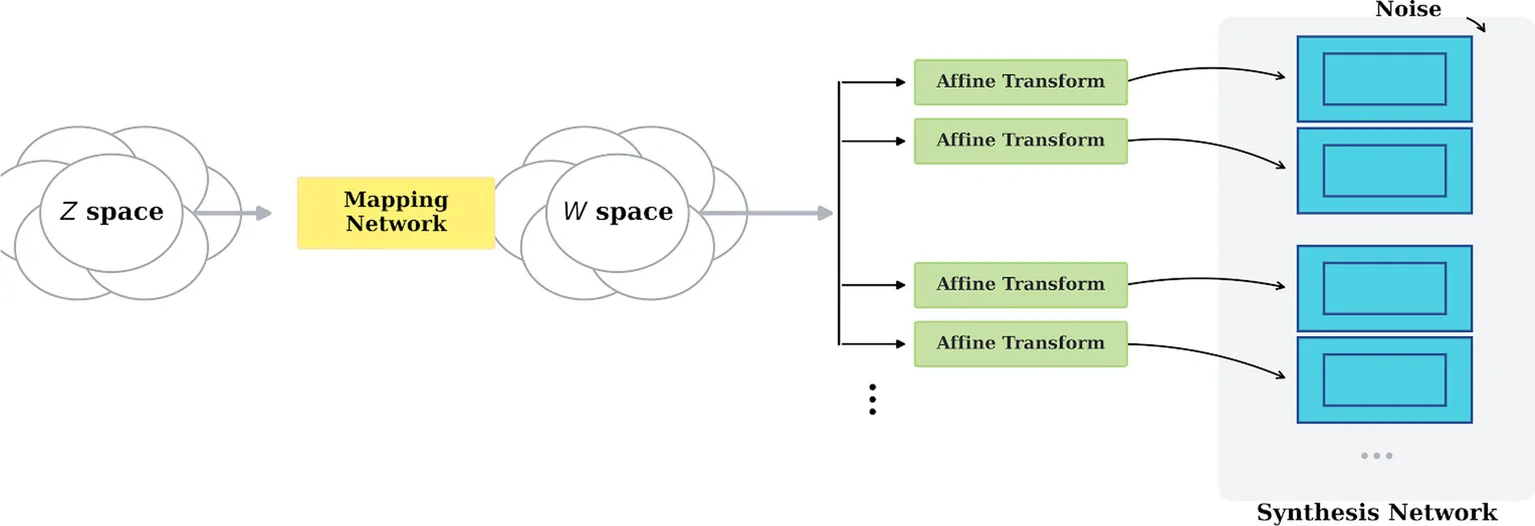
StyleGAN.

**Figure 8:**
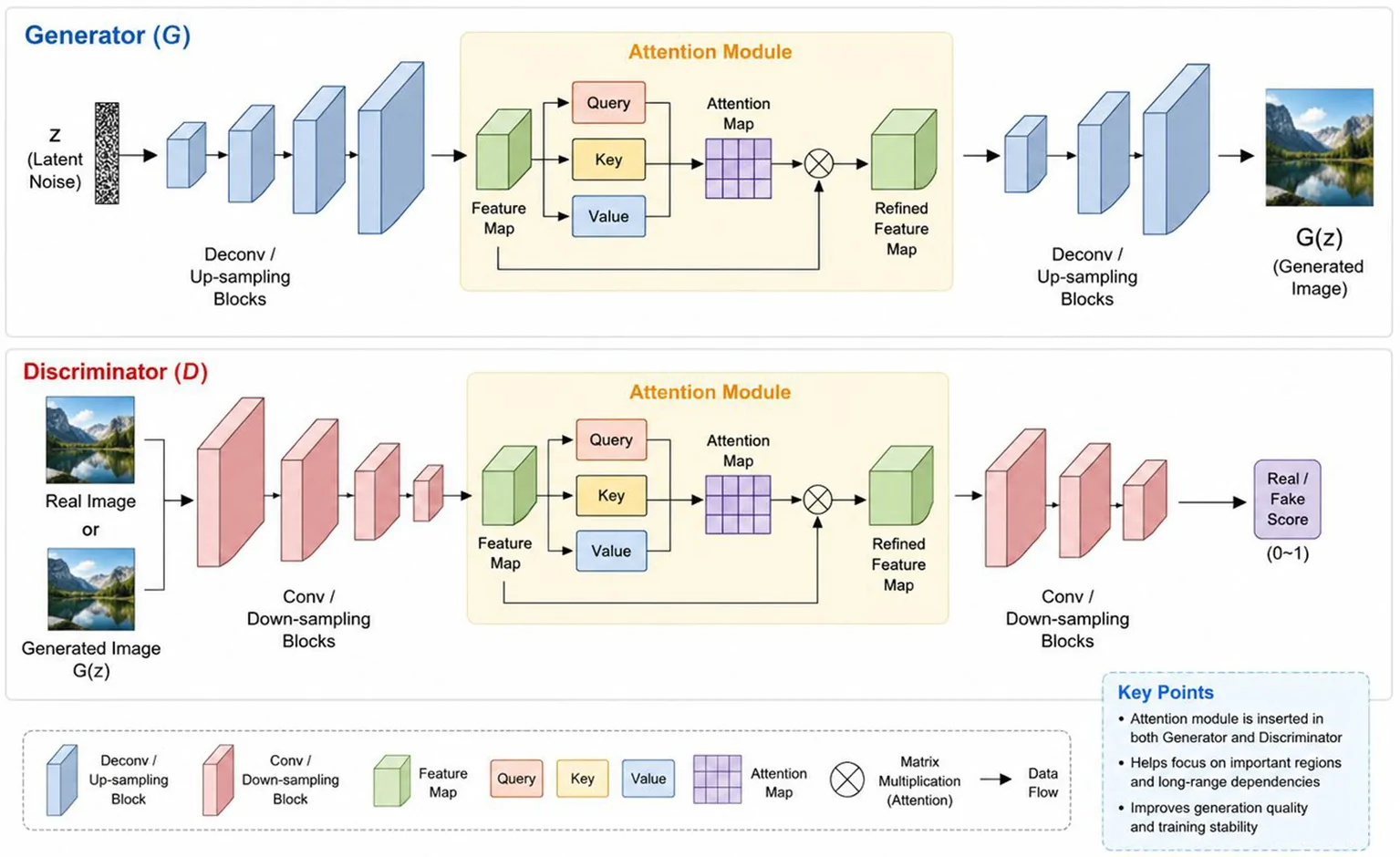
Attention-based GAN.

**Figure 9:**
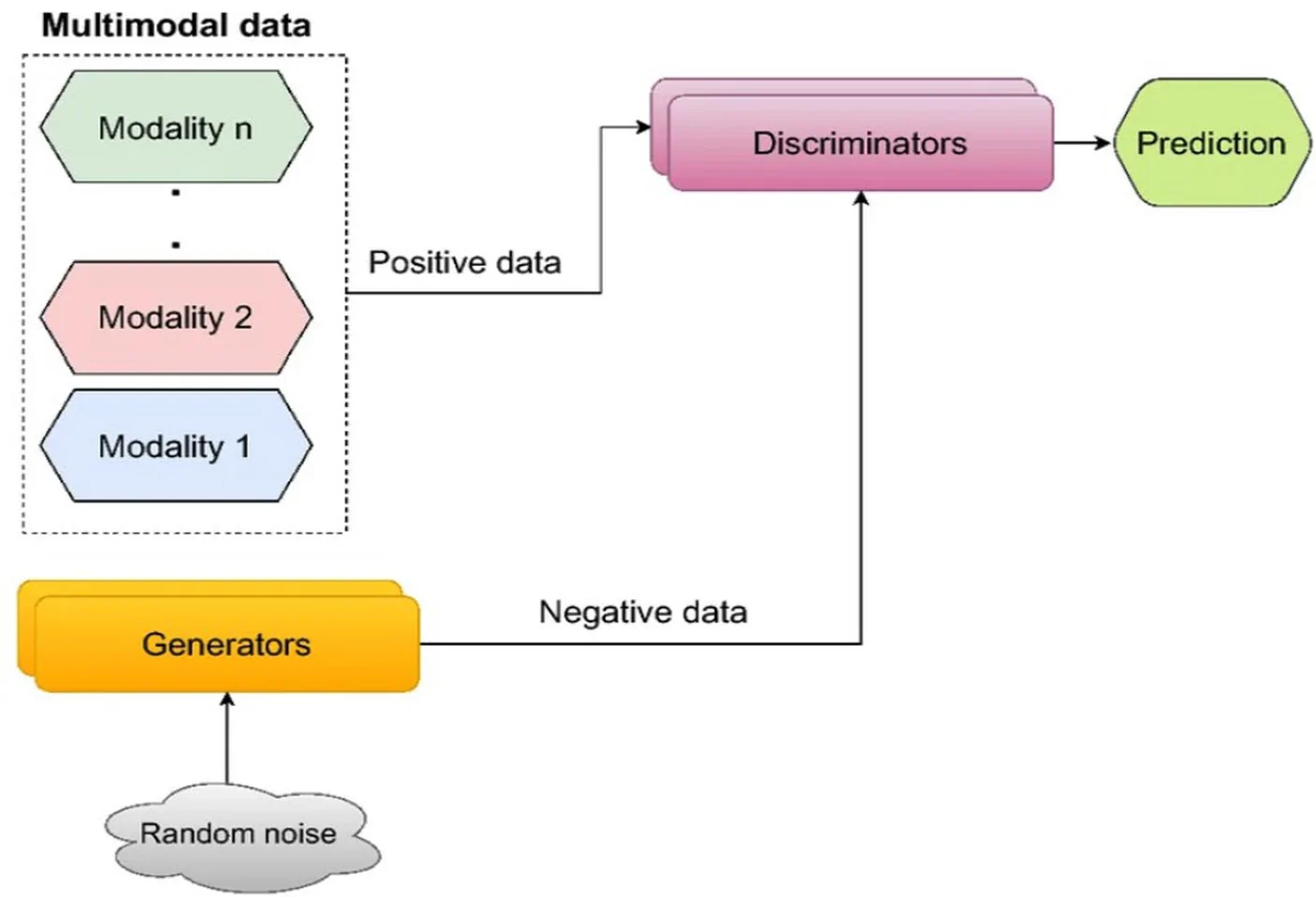
Multi-modal GAN.

**Figure 10:**
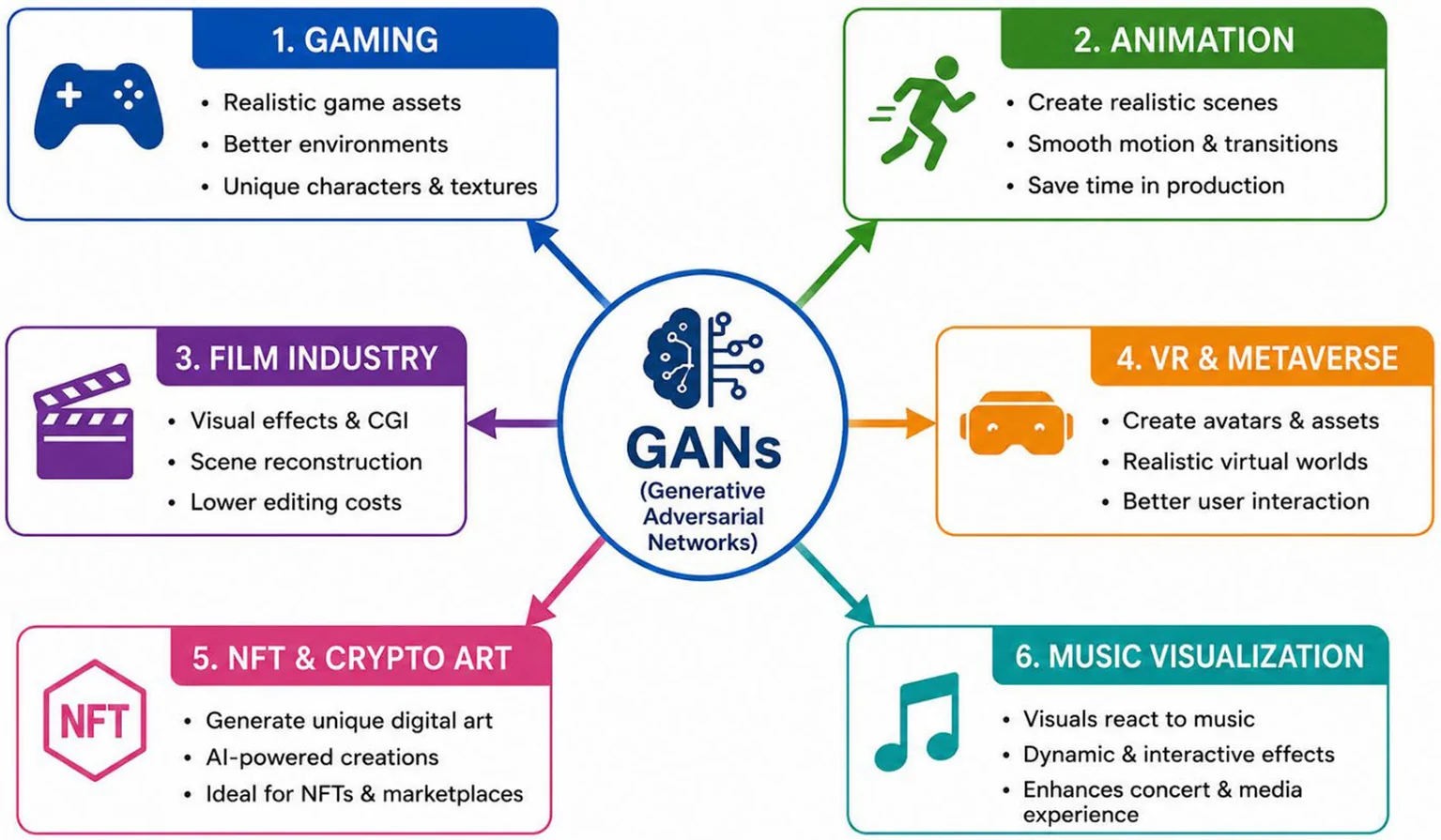
Application-based classification.

Previous research on GANs involves various models, datasets, applications, evaluation metrics, their benefits and drawbacks. It focuses on the various types of GANs such as DCGAN, StyleGAN, CycleGAN, and attention-based GANs in applications like digital art generation, text-to-image, super-resolution, style transfer, and deepfake detection. The analysis reveals that while GANs have achieved impressive success in terms of realism, scalability, and controllability, there are still challenges that need to be addressed, such as computational complexity, training instability, dependency on specific datasets, and lack of generalization in artistic and creative applications. While GANs have achieved impressive success in terms of realism, scalability, controllability, and a handful other metrics, there are still some challenges that need to be solved, such as computational complexity, training instability, dependency on a specific dataset, and lack of generalization (Figures 1–10).

### 7.1 Performance comparison

**Figure 11:**
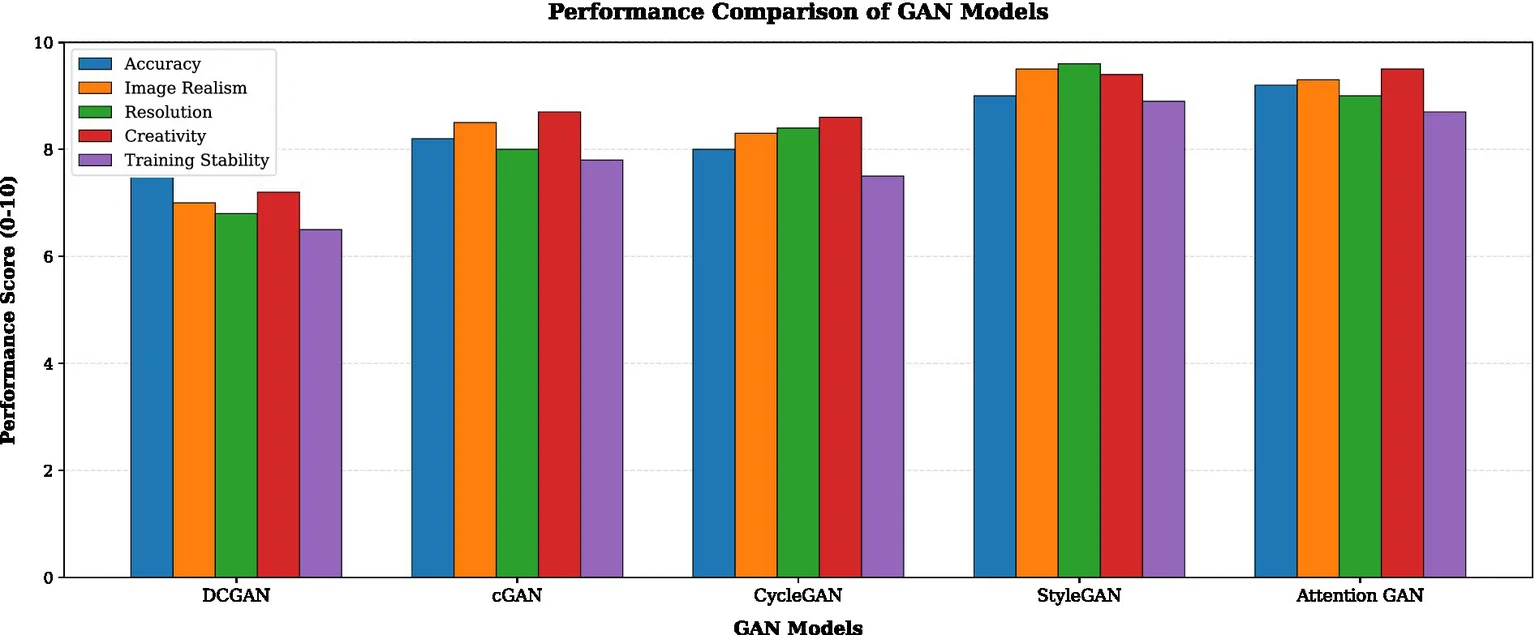
Performance comparison of GAN models.

We compare the results of various GAN models with respect to different criteria, such as accuracy, realism of images generated, image resolution, training stability, image creativity etc. as shown in Figure 11. The results show that the advanced models (StyleGAN, Attention GAN) perform better than the DCGAN model. StyleGAN has the best overall performance among the three models in terms of realism, resolution, and creativity, while Attention GAN is also performing well as it is able to capture the fine-grained details through attention mechanisms.

### 7.2 Computational complexity analysis

**Figure 12:**
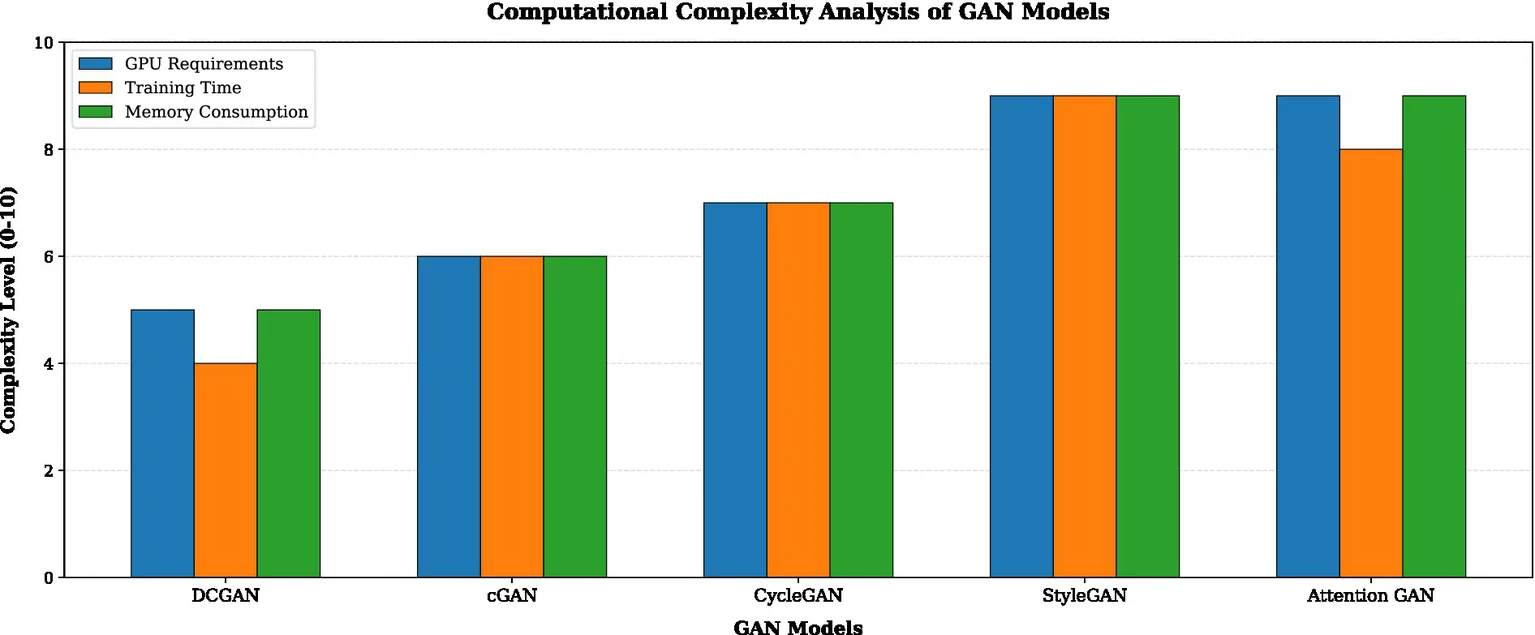
Computational complexity analysis of GAN models.

The computational complexity analysis of different GAN models is depicted in Figure 12 and it is clear that there are large differences in the demands for GPUs, training time and memory among the different models. The computational complexity of DCGAN is the lowest, while cGAN and CycleGAN have moderate resource needs. In comparison, StyleGAN and Attention GAN have the highest level of complexity due to their sophisticated architecture and their ability to produce high-quality images. The overall figure indicates that more complex GAN models require more computational power to be effectively trained and used.

## 8 Challenges and research gaps

While there has been substantial progress in the development of GANs for image generation, there are still numerous challenges and research gaps in technical, ethical, evaluative, and computational aspects. At a technical level, there are also ongoing problems like mode collapse, training instability, data scarcity, and overfitting that restrict the robustness of the models and their ability to generalize. However, there are ethical issues to consider, such as copyright infringement, ambiguity surrounding the ownership of AI-generated text, and the potential use of GANs for deepfake generation and misinformation. Cultural bias and dataset imbalance can result in unequal or unrepresentative outputs for various groups from a fairness standpoint. It is also difficult to evaluate because aesthetics is subjective, and there are no universally agreed-upon indicators of creativity and realism. GANs are expensive and energy-intensive in terms of computational requirements, as they demand a significant number of GPUs to generate images. Finally, there is a debate on the philosophical side of human creativity versus AI creativity, where a question that arises is whether there is originality, authorship, and whether it is true that AI can be a creative partner but not a competitor.

## 9 Future research directions

The text-to-image generation landscape is changing rapidly, moving from GAN-based methods to diffusion models, and, more recently, to flow matching models. The paradigm shift is due to the superior training stability of diffusion models, their enhanced semantic controllability, and the generation of highly diverse and detailed images, without the mode collapse problem that plagued GANs. Diffusion models, however, have significant computational cost and slower inference speeds, which may not be ideal for real-time interactive applications, where GANs still shine. Further studies are needed to explore hybrid architectures that can benefit from the best of both worlds: GANs for fast, real-time generation in games and interactive media, and diffusion models for high-quality, semantic-rich text-to-image generation in commercial art platforms. Furthermore, new flow matching models are promising in between, which may allow for quicker inference than diffusion but still provide stability in training. The future of digital art AI is probably not about the dominance of a single architecture over the other, but in the strategic integration of GANs, diffusion models, and flow-based approaches, each deployed according to the specific requirements of the artistic task, computational constraints, and user experience goals.

Further studies in art generation using the GAN are heading towards more sophisticated, efficient, and human-like systems. Key advancements include hybrid GAN-Transformer models and introducing diffusion models to improve image quality, stability, and scalability. Additionally, explainable creative AI is becoming a focus to increase transparency and understanding of generative processes. The idea behind real-time interactive art systems and personalized AI art generation is to empower users with more control and customization over their art creation. Additionally, the development of emotion-intelligent GANs relies on affective computing, which enables the generation of more expressive and contextually appropriate artwork. Furthermore, sustainable green AI methods are necessary to reduce energy consumption and computational expenses. Finally, the regulation of AI-generated content is becoming increasingly important due to ethical concerns, to prevent misuse and to ensure the responsible use of generative technologies.

## 10 Discussion

This section presents the main findings of the research, providing an overview of the major gains in realism, creativity and versatility of the applications across different architectures of the GANs. It also outlines some current research trends that are shaping the progress of generative models, such as the incorporation of attention mechanisms, the integration of transformers, and multimodal learning. Future potential directions are also discussed, such as diffusion–GAN hybrids, explainable AI frameworks, and real-time generative systems. Industry-wise, GANs are influencing the gaming sector, movie production, virtual reality and digital art, and have made a remarkable impact in the field of computer vision, machine learning and creative AI research. The overarching trends in the cross-disciplinary applications indicate that GANs are not merely about their technical applications, but about reshaping and reimagining the ways in which content is created and generated, and how humans interact with AI across different domains.

## 11 Conclusion

The aim of this study is to provide a broad overview of the entire field of image and artistic content generation with GANs, highlighting significant advances in realism, controllability, and creative synthesis in different GAN architectures and applications. The findings indicate that key advancements like attention mechanisms, transformer integration, and multimodal learning have significantly boosted semantic alignment, image fidelity, and user-initiated generation. The main contributions include the discussion of classification of GAN architectures, applications, datasets and evaluation methods, and the critical analysis of their pros and cons. Some of the gaps in the research identified were: evaluation standardization, ethical issues, computational efficiency and the interpretability of generative models. Overall, the findings indicate that hybrid generative models and systems involving collaboration between humans and AI will play a significant role in shaping the future of digital entertainment, paving the way for more immersive, adaptive, and responsible experiences.

## Data Availability

The original contributions presented in the study are included in the article/supplementary material, further inquiries can be directed to the corresponding author/s.
